# World Heart Federation Consensus on Transthyretin Amyloidosis Cardiomyopathy (ATTR-CM)

**DOI:** 10.5334/gh.1262

**Published:** 2023-10-26

**Authors:** Dulce Brito, Fabiano Castro Albrecht, Diego Perez de Arenaza, Nicole Bart, Nathan Better, Isabel Carvajal-Juarez, Isabel Conceição, Thibaud Damy, Sharmila Dorbala, Jean-Christophe Fidalgo, Pablo Garcia-Pavia, Junbo Ge, Julian D. Gillmore, Jacek Grzybowski, Laura Obici, Daniel Piñero, Claudio Rapezzi, Mitsuharu Ueda, Fausto J. Pinto

**Affiliations:** 1 Department of Cardiology, Centro Hospitalar Universitário Lisboa Norte, CAML, CCUL@RISE, Faculdade de Medicina, Universidade de Lisboa, Lisboa, Portugal; 2 Dante Pazzanese Institute of Cardiology – Cardiac Amyloidosis Center Dante Pazzanese Institute, São Paulo, Brazil; 3 Cardiology Department, Hospital Italiano de Buenos Aires, Buenos Aires, Argentina; 4 St Vincent’s Hospital, Victor Chang Cardiac Research Institute, University of New South Wales, Sydney, Australia; 5 Cabrini Health, Malvern, Royal Melbourne Hospital, Parkville, Monash University and University of Melbourne, Victoria, Australia; 6 Instituto Nacional de Cardiologia Ignacio Chavez, Ciudad de Mexico, Mexico; 7 Department of Neurosciences and Mental Health, CHULN – Hospital de Santa Maria, Portugal; 8 Centro de Estudos Egas Moniz Faculdade de Medicina da Universidade de Lisboa Portugal, Portugal; 9 Department of Cardiology, DHU A-TVB, CHU Henri Mondor, AP-HP, INSERM U955 and UPEC, Créteil, France; 10 Referral Centre for Cardiac Amyloidosis, GRC Amyloid Research Institute, Reseau amylose, Créteil, France. Filière CARDIOGEN; 11 Division of Nuclear Medicine and Molecular Imaging, Department of Radiology, Brigham and Women’s Hospital, Harvard Medical School, Boston, MA, USA; 12 Cardiac Amyloidosis Program, Cardiovascular Division, Department of Medicine, Brigham and Women’s Hospital, Harvard Medical School, Boston, MA, USA; 13 CV imaging program, Cardiovascular Division and Department of Radiology, Brigham and Women’s Hospital, Harvard Medical School, Boston, MA, USA; 14 Amyloidosis Alliance The Voice of the Patients, USA; 15 Hospital Universitario Puerta de Hierro Majadahonda, IDIPHISA, CIBERCV, Madrid, Spain; 16 Centro Nacional de Investigaciones Cardiovasculares (CNIC), Madrid, Spain; 17 Department of Cardiology, Zhongshan Hospital, Fudan University, Shanghai Institute of Cardiovascular Diseases, Shanghai, China; 18 National Amyloidosis Centre, University College London, Royal Free Campus, United Kingdom; 19 Department of Cardiomyopathy, National Institute of Cardiology, Warsaw, Poland; 20 Amyloidosis Research and Treatment Center, Fondazione IRCCS Policlinico San Matteo, Pavia, Italy; 21 Universidad de Buenos Aires, Argentina; 22 Cardiovascular Institute, University of Ferrara, Ferrara, Italy; 23 Department of Neurology, Graduate School of Medical Sciences, Kumamoto University, Japan

**Keywords:** Heart Failure, Amyloidosis, Transthyretin amyloid cardiomyopathy (ATTR-CM), diagnosis, treatment, patients’ perspective

## Abstract

Transthyretin amyloid cardiomyopathy (ATTR-CM) is a progressive and fatal condition that requires early diagnosis, management, and specific treatment. The availability of new disease-modifying therapies has made successful treatment a reality. Transthyretin amyloid cardiomyopathy can be either age-related (wild-type form) or caused by mutations in the TTR gene (genetic, hereditary forms). It is a systemic disease, and while the genetic forms may exhibit a variety of symptoms, a predominant cardiac phenotype is often present.

This document aims to provide an overview of ATTR-CM amyloidosis focusing on cardiac involvement, which is the most critical factor for prognosis. It will discuss the available tools for early diagnosis and patient management, given that specific treatments are more effective in the early stages of the disease, and will highlight the importance of a multidisciplinary approach and of specialized amyloidosis centres.

To accomplish these goals, the World Heart Federation assembled a panel of 18 expert clinicians specialized in TTR amyloidosis from 13 countries, along with a representative from the Amyloidosis Alliance, a patient advocacy group.

This document is based on a review of published literature, expert opinions, registries data, patients’ perspectives, treatment options, and ongoing developments, as well as the progress made possible via the existence of centres of excellence.

From the patients’ perspective, increasing disease awareness is crucial to achieving an early and accurate diagnosis. Patients also seek to receive care at specialized amyloidosis centres and be fully informed about their treatment and prognosis.

## 1. Introduction

Heart failure (HF) is a common syndrome in adults and one of the leading causes of cardiovascular morbidity and mortality. It significantly affects quality of life, and it is also associated with extremely high financial costs [1,2]. Worldwide, more than 64 million people have HF, with a prevalence of 1% to 3% in the general adult population and an incidence of 1 to 20 cases per 1,000 person-years [1]. These numbers increase as life expectancy increases. The burden associated with HF is rising due to major demographic changes, in particular population ageing [1].

As part of a global project, the World Heart Federation (WHF) has developed the Roadmap for Heart Failure, with the overall objective of reducing the burden of HF globally. This provides WHF Members and policy makers with a framework to guide initiatives within their national context, catalyze initial discussions, and plan a ‘call-to-action’ from key opinion leaders, thus raising awareness for the condition [3].

Around 50% of patients experience HF symptoms/signs in the presence of a preserved left ventricular ejection fraction, and half of these patients have increased left ventricular wall thickness [4,5,6]. Transthyretin amyloid cardiomyopathy (ATTR-CM), a restrictive cardiomyopathy resulting from the deposition of amyloid fibrils in the interstitial space of the heart muscle [7], commonly fits these characteristics. Having been considered a rare disease in the past, it has been increasingly identified as a much more frequent cause of HF than previously thought [8,9,10]. Transthyretin amyloid cardiomyopathy is a progressive and fatal condition if not diagnosed promptly. Management via specific treatments are now available and others are underway [3,11,12]. However, the condition remains misdiagnosed [13,14] and underdiagnosed [5] since awareness of the disease among clinicians and also in the community is far from desirable [15,16,17,18,19,20].

The WHF conducted an online survey to gather information on the availability and accessibility of health services for cardiac amyloidosis. The survey, consisting of 20 questions, was distributed to WHF member organizations from January 12 to January 30, 2023. A total of 41 participants from 20 countries completed the survey. The results can be found in Supplementary Table 1. Around 70% of the participants were practicing cardiologists. It is worth noting that over 70% of the participants agreed that clinicians generally have limited awareness of cardiac amyloidosis. Despite the majority of participants treating only a small number of cardiac amyloidosis patients each year (one to nine patients), several were familial with the signs of cardiac amyloidosis and recognized the importance of early diagnosis. According to the survey, suspicion of the diagnosis primarily occurs at the secondary healthcare level (56%). However, the diagnosis itself is typically made at the tertiary care level.

Transthyretin amyloidosis can primarily manifest as heart involvement, particularly in the wild-type form (ATTRwt), associated with aging and a major cause of HF in the elderly. On the other hand, ATTR-CM may be part of a systemic disease where insoluble amyloid fibrils accumulate in various organs [19], resulting in diverse clinical manifestations as observed in the genetic (hereditary) forms (ATTRv, v for variant). This heterogeneity poses challenges for diagnosis and delays it. In both subtypes of ATTR-CM, cardiac involvement is the main determinant of prognosis, quality of life, and mortality [9].

### Why this consensus document?

The global incidence of ATTR-CM is rising [21] due to several factors: 1) increased utilization of imaging techniques enabling early and non-invasive diagnosis in most cases; 2) expanded availability of genetic testing; 3) published diagnostic algorithms and consensus recommendations promoting disease awareness [21,22,23,24,25,26]; and 4) possibility of targeted treatments that can positively influence prognosis [21,22,23,24,25,26]. With an aging population, HF, the most common manifestation of ATTR-CM, and aortic stenosis (AS), a common valvular disease of the elderly frequently coexist [22,23], will become more prevalent, and ATTRwt will be diagnosed more often because it is not uncommon in these settings. Diagnosing ATTR-CM is challenging for clinicians, as it can mimic symptoms of other common diseases. Early detection often relies on vigilant physicians recognizing potential ‘red flags.’

This document aims to: 1) provide a comprehensive review of ATTR amyloidosis, particularly cardiac involvement; 2) present current tools and effective pathways to early diagnosis, considering the high misdiagnosis rate of approximately 40–50% in ATTR-CM cases [13,24]; 3) underscore the significance of registries and clinical trials for gathering essential data and offering opportunities to patients; 4) prioritize patients’ needs, including treatment accessibility, and the role of Patient Advocacy Groups; and 5) highlight the importance of multidisciplinary patient management, emphasizing the key role of specialized amyloidosis centres. By increasing awareness of this condition, which affects more individuals than previously recognized, it is essential to prompt the scientific community to prioritize early diagnosis and treatment.

## 2. Methods

To achieve the proposed goals, the WHF organized a multidisciplinary panel of expert clinicians in transthyretin amyloidosis to plan and develop this consensus document.

Experts were selected by the WHF leadership, based on several requirements: all experts had extensive experience in the management of cardiac amyloidosis, with scientific contributions in the areas of amyloidosis and/or cardiomyopathies, including previous participation in consensus or expert documents, guidelines, and other scientific production; several invited experts are representatives of relevant clinical societies, and most are representatives of national amyloidosis reference centres in their countries. The group was recruited internationally from different regions of the globe and includes 18 medical experts from 13 countries, representing four continents (Europe, Americas, Asia, and Australia). Their expertise covers the areas of Cardiology, Clinical Epidemiology and Ageing, Imaging (echocardiography, cardiac magnetic resonance) and Nuclear Medicine, Internal Medicine, Nephrology, and Neurology. In addition, a Patients’ representative (Amyloidosis Alliance) was also invited to contribute to the document and participate as a co-author. The document was developed after a first roundtable meeting (mixed format, face-to-face and virtual), and rounds of feedback.

## 3. Transthyretin amyloidosis

### 3.1. The disease

#### 3.1.1. Definition and general considerations

Systemic amyloidosis includes a broad spectrum of diseases that result from misfolding, aggregation, and deposition of proteins in several organs. These heterogeneous conditions are named according to their amyloidogenic precursor protein. For example, in transthyretin amyloidosis (ATTR) the amyloid (A) fibril is formed by transthyretin (TTR), a tetrameric transport protein mainly synthesized in the liver, in the choroid plexus and by the retinal pigment epithelial cells [7,27]. Under particular conditions, only partially characterized, TTR may become unstable, misfold, and deposit in the form of insoluble amyloid fibrils in the extracellular space of organs and tissues [7]. Cardiac amyloidosis occurs when fibrils accumulate in the heart, often resulting in restrictive cardiomyopathy [25,26,28].

**Figure 1:**
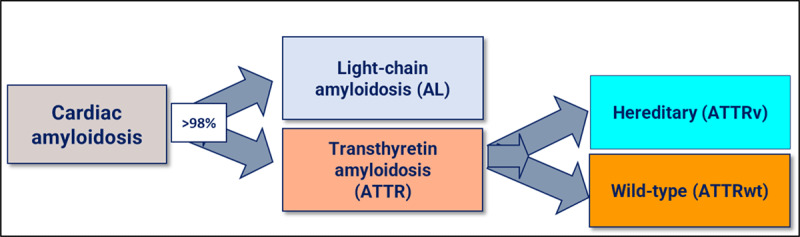
The most frequent amyloidosis subtypes that affect the heart.

TTR is not the only protein that may deposit as amyloid in the heart. The other most common form of cardiac amyloidosis includes monoclonal immunoglobulin light chain disease (AL-CM) (Figure 1). In the latter, a plasma cell clone infiltrates the bone marrow and produces monoclonal light chains that deposit in tissues, including the heart in approximately 50% of cases. Amyloid light-chain (AL) is a rare hematologic disease with multisystemic involvement and an incidence of 10–12 per million person-years [29]. While the amyloid fibril deposition in AL-CM and ATTR-CM can manifest systemically, it is the cardiac involvement that determines prognosis [9,30].

Monoclonal immunoglobulin light chain disease may be either genetic, associated with mutations in the TTR gene or sporadic, related to age (ATTRwt). Although these two different diseases (or subtypes, as they are commonly considered) have important differences, heart involvement is indistinguishable between them by clinical findings or investigation characteristics [7,9,30,31].

However, hereditary ATTR-CM has a more heterogeneous multisystem presentation compared to ATTRwt, as the deposition of ATTR can occur in multiple organs beside the heart, most commonly the peripheral nervous system (ATTR polyneuropathy [ATTR-PN]) and autonomic nervous system, musculoskeletal system, eye, kidney, and gastrointestinal tract [30,32,33,34]. Often, the absence of a family history of amyloid disease, and the presence of a predominant cardiac phenotype, are among the factors that contribute to a long delay in diagnosis and reason for the patient to visit several physicians of different specialties before the correct diagnosis is made [35,36].

Transthyretin Amyloidosis Cardiomyopathy has been considered a rare disease. However, in the last 10 years, mainly due to advances in non-invasive cardiac imaging and diagnostic methods, the condition is currently considered much more common than previously thought, with a substantial increase in the number of diagnosed patients [23,28,29,37,38,39,40,41].

Nonetheless, the exact epidemiology of ATTR-CM is still unknown, and a true estimate of prevalence is difficult [26,42,43]. In a series of 56 autopsied hearts from unselected adults more than 75 years of age, 43% showed cardiac amyloid deposition, and 50% of those had ATTR [44].

ATTR amyloidosis is observed in geographically dispersed populations. Different registries have been implemented aiming to better characterize ATTR patterns and gather epidemiological data [37,38,39,45,46]. The THAOS (The Transthyretin Amyloidosis Outcomes Survey) registry, established in 2007 (NCT00628745), is the largest, global, longitudinal observational study of patients with ATTR amyloidosis. The registry was designed to evaluate overall survival in patients, better understand genotype-phenotype relationships and the natural history of ATTR amyloidosis, and the effects of available treatments on the progression of the disease [47,48]. The most recent update is a 14-year global overview and includes 3,779 symptomatic patients and 1,830 asymptomatic carriers of pathogenic TTR gene mutations from 84 study sites in 23 countries, representing the largest analysis of ATTR amyloidosis to date [48]. Among symptomatic patients at enrolment, 40.7% presented a predominantly cardiac phenotype, 40.1% had a predominantly neurologic phenotype, and 16.6% were identified with a mixed phenotype [48].

#### 3.1.2. Characteristics of the two subtypes of ATTR-CM

The two subtypes of ATTR-CM have different characteristics regarding pathophysiology, epidemiology, demographic phenotypes, and prognosis.

##### 3.1.2.1. Genetic ATTR-CM

Over 140 different TTR gene mutations have been identified, but only some are associated with most ATTRv amyloidosis cases [49,50]. Most mutations causing ATTRv amyloidosis are gain of function mutations with autosomal dominant inheritance. However, penetrance and expressivity are highly variable among the diverse variants, even within the same family [51,52,53].

Genetic ATTR disease presents an age of onset ranging from the second to the ninth decade of life and p.Val50Met (previously designated Val30Met) is the most recognized TTR gene mutation [48] and the most prevalent genotype among all THAOS patients (49.6%). Its early onset phenotype, typically causing ATTR polyneuropathy (also referred to as familial amyloidosis polyneuropathy) has endemic focus in Portugal, Sweden, and Japan [48]. Other mutations, particularly those more commonly associated with ATTR-CM, manifest as late onset phenotypes. These include among others the late-onset form associated with the p.Val50Met mutation, and the p.Val142Ile mutation (previously designated V122I), which occurs more commonly in African-American, Western, African, and Hispanic populations, described with an estimated prevalence of 4% in the United States (US) [26].

The p.Ala117Ser TTR variant in China and Taiwan and the p.Thr80 Ala mutation, occurring mostly in patients of Irish descent [54] (and often manifesting as a mixed neuropathic and cardiomyopathic phenotype [26]) have been considered founder mutations in these regions [30].

Although the phenotypic heterogeneity of the disease contributes to challenging the diagnosis [54,55,56,57] some data from the THAOS analysis emerged regarding the genotype-phenotype relationship in what concerns the predominant cardiac, neurologic, or mixed phenotypes.

###### Cardiac phenotype

Cardiomyopathy is more common in late-onset than in early-onset Val50Met [30]. Non-Val50Met ATTR-CM amyloidosis has been particularly associated with four variants designated as ‘cardiac mutations’ [48]: p.Val142Ile [58], Leu131Met [59], p.Thr80 Ala [60], and Ile88 Leu [61]. These ‘cardiac mutations’ are present in 384 (74% male) out of the 3,779 symptomatic patients included in the THAOS registry, with a mean age of 63.5 years at the onset of ATTR amyloidosis symptoms [48]. The predominant cardiac phenotype associated with these mutations includes cardiomyopathy with left ventricular hypertrophy (LVH) and HF (affecting 92.2% of the patients with cardiac mutations included in THAOS) [48,53].

###### Neurologic phenotype

Peripheral polyneuropathy (PN) is frequently associated with the p.Val50Met mutation, being the most common phenotype of ATTRv: in the THAOS registry, 72.8% of patients with p.Val50Met had a predominantly neurologic phenotype at enrolment [48]. Early onset ATTRv-PN associated with Val50Met is common in Portugal and Japan and is mainly characterized by a high-penetrance progressive axonal length-dependent sensorimotor and autonomic polyneuropathy. In the THAOS registry, out of 826 patients included with the early onset p.Val50Met (mean age at onset 33.1 years), cardiac involvement affected 24.2% of patients [48].

The late onset ATTRv-PN amyloidosis is characterized by progressive axonal sensorimotor neuropathy affecting all sensory modalities, variable penetrance, and lack of family history [39]. Cardiac involvement affects 39.6% of patients with the late-onset p.Val50Met mutation [48].

###### Mixed phenotype

Mixed phenotypes combine features of both cardiomyopathy and polyneuropathy [9]. Different factors contribute to the natural history of patients with a mixed phenotype, including age of onset and primary phenotype. Among patients with ‘cardiac mutations’, 17.2% have been considered as having a mixed phenotype. However, the percentage is likely to be much higher, on the basis of a study showing that individuals of African descent carrying the p.Val142Ile mutation are at increased risk of polyneuropathy [62]. Moreover, 10% of symptomatic patients with ATTRwt also present a mixed phenotype [61].

###### Prognosis

The prognosis of ATTRv varies according to the different mutations and the phenotype. The median overall survival following diagnosis in untreated patients was reported to be 4.7 years [63]. However, there is a clear variation in survival concerning genotype in ATTRv amyloidosis, with median survival in the range of 8 to 10 years for patients with ATTRv amyloidosis and polyneuropathy [9]. In patients presenting with cardiomyopathy-predominant phenotype, survival is further reduced to 3.4 years [60,63], and death is usually due to progressive HF or life-threatening cardiac arrhythmias [64,65].

##### 3.1.2.2. Wild-type ATTR-CM

Wild-type ATTR-CM may have an onset at 60 to 65 years of age, although its prevalence increases at older ages, and it affects predominantly males [9,31,66].

The prevalence of ATTRwt-CM is unknown, but it is surely much more frequent than previously thought, having been diagnosed in several clinical contexts involving the elderly as already mentioned [8,23,67,68,69,70,71,72,73,74,75]. ATTRwt patients can also develop neurologic complications including polyneuropathy [66,76].

The median survival for untreated patients with ATTRwt-CM is approximately five years from diagnosis [26,77]. The disease may be associated with sudden deaths among the elderly [78].

### 3.2. ATTR and the heart

#### 3.2.1. Clinical manifestations

The clinical spectrum of cardiovascular involvement in ATTR-CM varies widely, ranging from asymptomatic to severe manifestations. There may be pronounced thickening of left ventricular (LV) wall with restrictive cardiomyopathy and progressive HF [22]. Autonomic dysfunction associated with ATTR-CM can cause orthostatic hypotension and syncope [79].

Fatigue is a common debilitating symptom and can be challenging to measure, especially in older individuals. Heart failure is the most common clinical presentation for ATTR-CM patients [9]. Its progression is usually gradual, starting with exercise intolerance and worsening dyspnoea on exertion, pulmonary congestion, orthopnoea, and paroxysmal nocturnal dyspnoea, advancing to overt biventricular HF symptoms and signs, with peripheral oedema, early satiety, and abdominal bloating. Left ventricular ejection fraction (LVEF) is usually preserved although it may decrease in later stages. Transthyretin Amyloidosis Cardiomyopathy is frequently misdiagnosed as other conditions such as hypertensive heart disease, hypertrophic cardiomyopathy (HCM), or even AS, particularly in the elderly. Up to 15% of patients with AS may have ATTR-CM and in the subpopulation with low-flow, low-gradient pattern it can rise to 30% [23,80,81]. Cardiac amyloid deposition can affect various cardiovascular structures, including the myocardium and valves. Amyloid deposition in the LV typically starts from the base to the apex, resulting in increased biventricular wall thickness and stiffness. These conditions will ultimately lead to pseudohypertrophic restrictive cardiomyopathy with severe impairment of LV diastolic function and compromised longitudinal systolic function that commonly (although not always) spares the ventricular apex [82,83].

The natural history of ATTR-CM also includes rhythm disturbances [9], particularly atrial fibrillation (AF) and conduction system disease [54]. The prevalence of AF among ATTR-CM patients has been estimated to be 44% to 70% [84,85,86,87], and symptomatic bradycardia and/or advanced atrioventricular (AV) block needing permanent pacemaker implantation are recognized disease complications [84,87]. The involvement of the autonomic nervous system [88] may lead to a progressive decline in blood pressure and intolerance to previous drugs for hypertension treatment or HF condition.

Electrocardiogram (ECG) and echocardiogram (echo) are practical tools that can help raise suspicion of cardiac amyloidosis [89].

#### 3.2.2. The electrocardiogram

ECG patterns may be suggestive of amyloid heart disease, although sensitivity and specificity are low [90]. However, ECG abnormalities may act as ‘red flags’ for the condition in certain clinical scenarios (e.g., HF, LVH of unknown cause).

The existence of increased LV wall thickness in the presence of low-voltage QRS is highly suggestive of cardiac amyloidosis and can differentiate it from LVH due to hypertension or HCM [9]. However, only 25% to 40% of patients with ATTR-CM meet low voltage criteria [9,91,92]. In addition, the low voltage may reflect the increasing accumulation of amyloid protein over time [93,94] and, as such, it is a relatively late finding of ATTR-CM and may not be useful for early identification. The lack of progression of the R wave (pseudo-infarction pattern) in the anterior precordial leads was reported in more than half of affected patients [13,95]. A proportion of patients (12% to 25%) with amyloid cardiomyopathy have a LVH pattern on ECG [13,96]. Conduction defects, such as various degrees of AV block, fascicular block, intraventricular conduction delay, and bundle branch blocks, are also frequently observed [10,81] ventricular arrhythmias including ventricular tachycardia or fibrillation may be a cause of sudden death in ATTR-CM patients.

Machine learning techniques have been used to develop an ECG-based tool from a detailed electroanatomic mapping of patients with cardiac amyloidosis. The derived ECG algorithm has proven helpful in providing an initial suspicion of the condition [97].

#### 3.2.3. The echocardiogram

**Figure 2:**
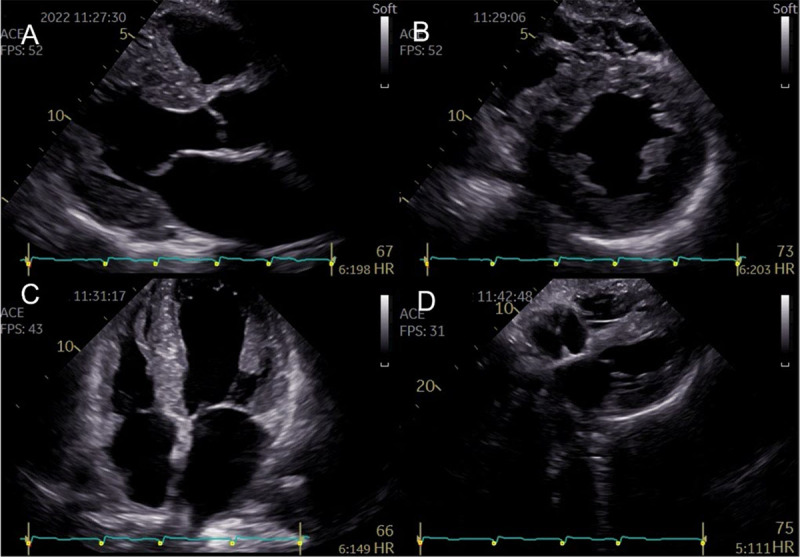
Representative two-dimensional echocardiographic findings of cardiac amyloidosis in a patient with ATTR-CM. **(A)** Parasternal longitudinal view **(B)** Short axis view **(C)** Apical 4-chamber view **(D)** Subcostal view Concentric left ventricular and right ventricle free wall hypertrophy, thickened interatrial septum, and atrioventricular valves. (Images courtesy of Centro Hospitalar Universitário Lisboa Norte, Lisboa, Portugal).

**Figure 3:**
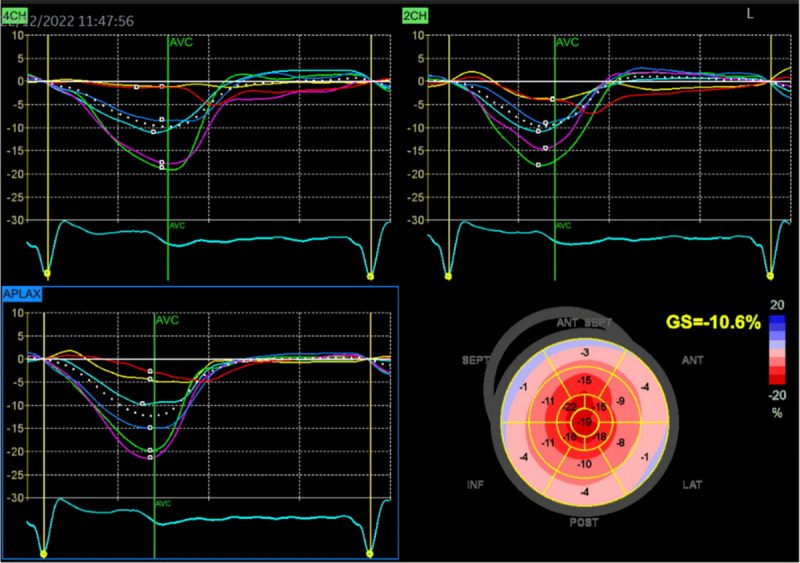
Two-dimensional speckle-tracking strain imaging echocardiography of a patient with ATTRwt-CM (the same patient as in Figure 2). Reduced left ventricular longitudinal strain in the middle and basal segments with relatively preserved strain in the apex (i.e., apical sparing) is observed. A bull’s eye is shown in the lower right panel (Images courtesy of Centro Hospitalar Universitário Lisboa Norte, Lisboa, Portugal).

Echo is a widely available tool that often provides the first clues to the presence of cardiac amyloidosis. Diagnostic clues include increased LV wall thickness (typically ≥ 1.2 cm) with an asymmetrical (most common) [98] or symmetrical pattern, a non-dilated LV, the ‘granular sparkling’ appearance of the myocardium, AV valve/right ventricle free wall/interatrial septum thickening, pericardial effusion, diastolic dysfunction, decreased mitral annular systolic velocity (s’), biatrial enlargement, and decreased global longitudinal strain (GLS) with relative apical sparing [99] (Figures 2, 3, and Video 1).

**Video 1:**
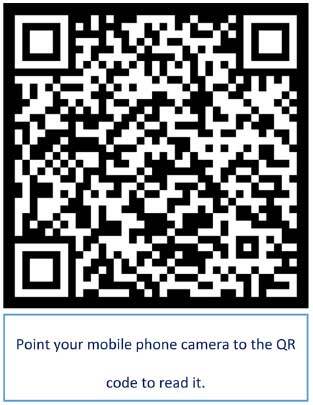
37-second recording in real-time showing typical echocardiographic features of a patient with ATTR-CM (the same patient as in Figures 2 and 3): concentric left ventricular hypertrophy, interatrial septum thickening, biatrial enlargement, and decreased global longitudinal strain with relative apical sparing. There is mild mitral and aortic regurgitation. The ejection fraction of both the left (LV) and right ventricle (RV) is preserved (4D analysis, LV in red, RV in blue). The patient gave informed consent for imaging and video use. https://dulcebritocardiologista.com/wp-content/uploads/2023/01/Amyloidosis-Final.edited.video_.mp4

This phenomenon of a longitudinal strain gradient showing relative preservation of function at the apex, as well as substantial impairment at the mid-segments and basal segments, is a very constant and characteristic finding in patients with cardiac amyloidosis [100]. Global longitudinal strain is a more sensitive measure of myocardial dysfunction than ejection fraction [101,102,103,104,105].

Several echo parameters and indices have high specificity to support a potential diagnosis of amyloidosis in the appropriate clinical setting, adding value to the conventional echo criteria, such as an increased E/E’ ratio and a low myocardial contraction fraction [106]. The multitude of abnormal echo parameters described in cardiac amyloidosis led to the development of multi-parametric scores for the diagnosis [107,108,109,110]. These may be of particular importance in early disease, when the echo changes are subtle and less specific [107,108]. Scores help to objectify the echo parameters and a more widespread use could decrease delays in diagnosis and treatment [111].

Machine learning techniques offers a promising approach to detect the variable and diverse echo features associated with cardiac amyloidosis, even in early stages, and alert clinicians to consider further testing. This was already done in a large multicentre study using machine learning techniques of both ECG and echo in cardiac amyloidosis [112]. The model was capable to detect cardiac amyloidosis by echo up to one year before diagnosis, and additionally, it was able to discriminate it from other conditions causing LVH [112].

#### 3.2.4. Cardiac magnetic resonance imaging

**Figure 4:**
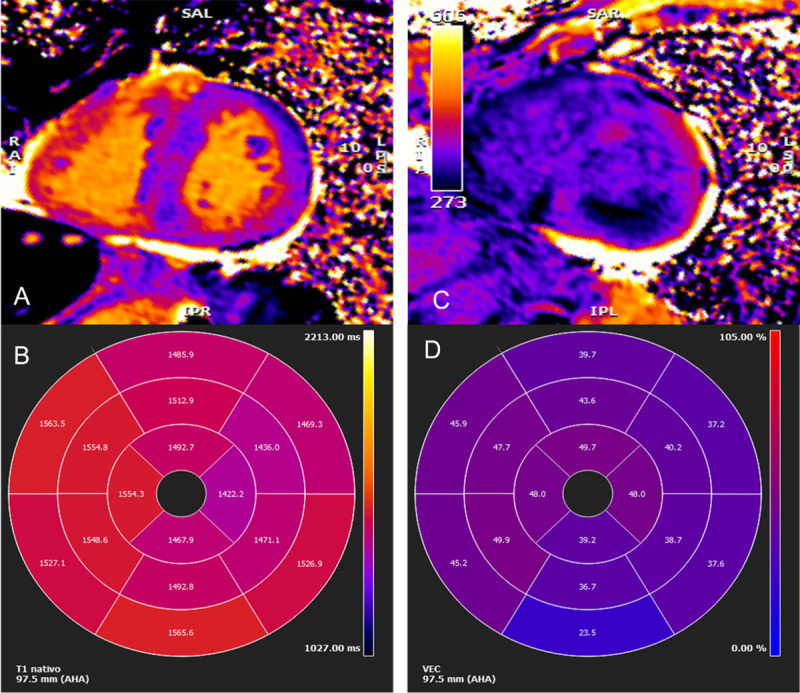
Cardiac magnetic resonance imaging. **(A)** Myocardial native T1 mapping (short axis) **(B)** Native T1 global polar map with abnormally increased T1 relaxation time (myocardial T1 values: 1,512±72 ms) **(C)** Post-contrast T1 **(D)** Global extracellular volume map, calculated from both native and post-contrast T1 myocardial values: in this case high above normal: 44±9%. (Images courtesy of Lusíadas Hospital, Lisboa, Portugal).

**Figure 5:**
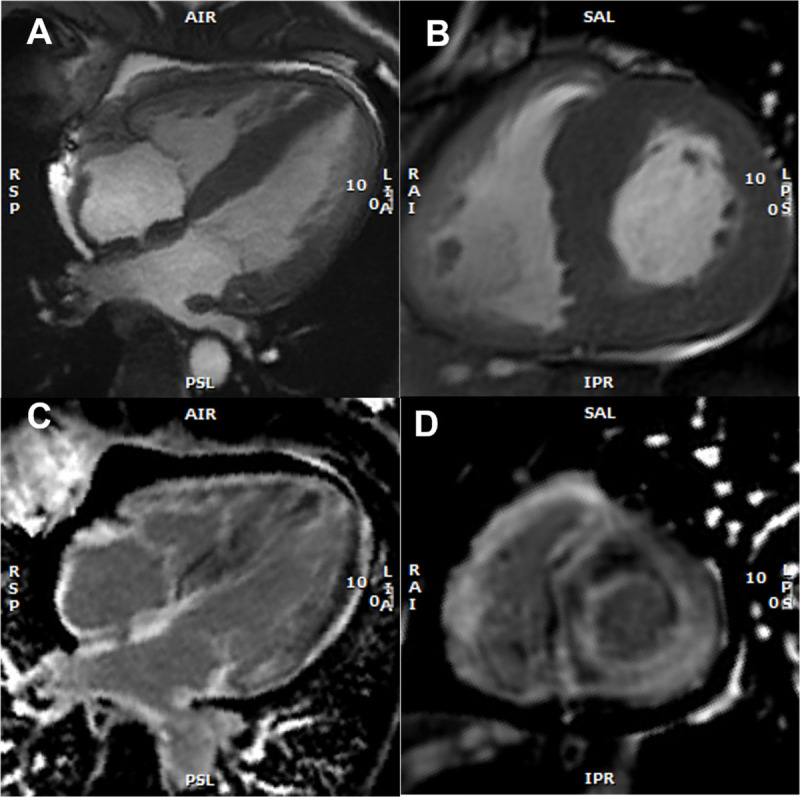
Cardiac magnetic resonance imaging findings with representative examples. **(A)** Cine: four-chamber view – *SSFP (steady-state free precession)* acquisition, depicting asymmetric left ventricular (LV) hypertrophy, inter-atrial septum thickening, and mild pericardial effusion **(B)** Short axis view – SSFP, asymmetric LV hypertrophy **(C)** Late gadolinium enhancement (LGE) at the 4-chamber view with subendocardial LGE at the left ventricle, atrial Wall, and inter-atrial septum **(D)** Short axis view, showing subendocardial and subepicardial LGE at the left ventricle and also at the right ventricle. (Images courtesy of Lusíadas Hospital, Lisboa, Portugal).

In selected patients, cardiac magnetic resonance (CMR) may be used as an initial evaluation tool for cardiac amyloidosis or as supplementary method when suspicion is based on echo imaging. Cardiac magnetic resonance is particularly useful when ultrasound yields poor acoustic window and provides valuable information regarding tissue characterization of the myocardium, especially when gadolinium-based contrast agents are employed, offering high diagnostic accuracy for cardiac amyloidosis [103,104,113]. The expansion of the extracellular volume (ECV), abnormal gadolinium contrast kinetics, and diffuse late gadolinium enhancement (LGE) are notable CMR features of cardiac amyloidosis [103,104,113] (Figures 4 and 5). Initially, amyloid cardiomyopathy presents a typical pattern of diffuse subendocardial LGE, which may progress to transmural involvement in later stages of the disease [113]. The degree of myocardial infiltration by amyloid correlates well with the patterns of LGE [98], and showed a clear relationship with mortality at 24 months in a large study [98,114]. Right ventricle LGE was also a predictor of a worse prognosis [115]. T1 mapping is a quantitative method that allows the measurement of cardiac amyloid infiltration from early phases without LGE to extensive diffuse transmural involvement [115,116]. Prior to contrast agent administration, T1 mapping can be used to measure the intrinsic signal from the myocardium (native myocardial T1) [98,114]. By combining these measurement with studies conducted after the administration of gadolinium-based contrast agent, it becomes possible to calculate the myocardial ECV, which represents, the proportion of extracellular space occupied by amyloid deposits [99]. Native T1 and ECV are elevated in patients with ATTR-CM or AL-CM [99,117] and have both been extensively validated as indicators of cardiac amyloid infiltration [116,118], correlating with infiltration measured using other techniques such as ^99 m^Tc-3,3-diphosphono-1,2-propanodicarboxylic acid (^99 m^Tc-DPD) scintigraphy [116,119]. Extracellular volume is a predictor of prognosis in patients with ATTR-CM [116]. However it is important to note that CMR is neither necessary nor sufficient for establishing the diagnosis of cardiac amyloidosis as a standalone test and cannot distinguish between ATTR-CM and AL-CM [120,121].

Cardiac magnetic resonance can also measure myocardial perfusion with fully automated myocardial blood flow mapping [122]. Intramyocardial vessels are frequently infiltrated by amyloid, resulting in impaired vasodilatation, which can cause global myocardial ischemia. Levels of cardiac biomarkers (troponin T and NT-proBNP) are known to be constantly elevated in patients with cardiac amyloidosis [123], and a decreased myocardial perfusion may contribute to these increases. It is worth noting that these biomarkers play a crucial role in measuring the response to new amyloid therapies that directly target amyloid deposits.

#### 3.2.5. Nuclear medicine imaging

##### 3.2.5.1. The role of diphosphonate scintigraphy in the non-invasive diagnosis of ATTR

Cardiac nuclear scintigraphy using bone avid radiotracers is the sole imaging method capable of accurately diagnosing ATTR-CM without the requirement of invasive cardiac biopsy. This modality becomes relevant when serum and urine tests for AL amyloidosis yield negative results [22,31,102,124,125].

Three technetium ^99 m^(Tc)-labeled phosphate-based bone avid scan radiopharmaceuticals are useful to detect cardiac ATTR, including hydroxymethylene diphosphonate (HMDP), pyrophosphate (PYP) and DPD. Overall, their diagnostic accuracy appears equivalent [126]. Tc-99 m methylene diphosphonate (MDP) is not recommended as its sensitivity is significantly lower compared to the other agents [103,127].

**Figure 6:**
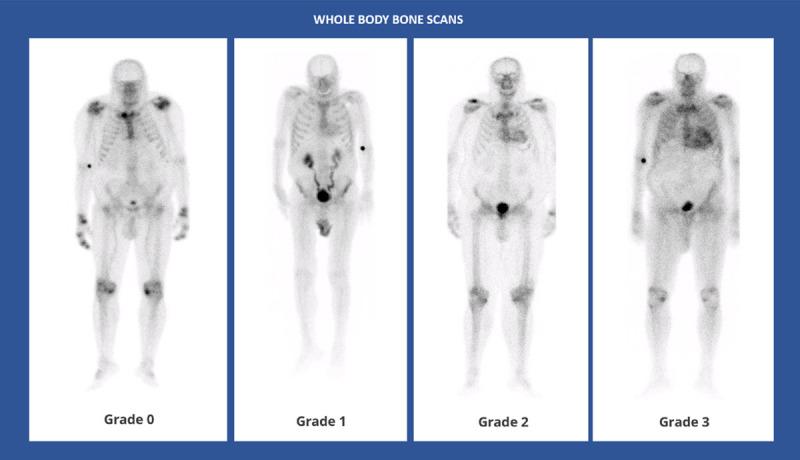
Whole body anterior planar views performed 3 hours post-injection of Tc99 m-DPD. Four different patients with different Perugini visual scores. (Images courtesy of Lisbon Medical School, Faculdade de Medicina da Universidade de Lisboa, Portugal).

Quantification of the intensity of radiotracer uptake by the heart is important in the diagnosis of ATTR-CM using bone scintigraphy. The intensity of retention of bone-avid radiotracers in the heart can be interpreted by semiquantitative visual analysis, by grading myocardial uptake in relation to rib uptake on planar or single-photon emission computed tomography (SPECT) images, and by quantifying radiotracer uptake using the heart-to-contralateral lung (H/CL) ratio [128]. Perugini et al. classified cardiac amyloid uptake based on a simple visual scoring system of the delayed (3h) planar image, in which a grade of 0 means no cardiac uptake, a grade of 1 means mild cardiac uptake (less than in bone), a grade of 2 means cardiac uptake with intensity similar to bone rib uptake, and a grade of 3 means substantial cardiac uptake (with a weak or no signal evident in bone) [129,130] (Figure 6).

Diagnostic criteria for positive planar scintigraphy include a Perugini score ≥2 [22,31,102,124,125], and/or a heart/contralateral (HCL) chest ratio ≥1.5 on a 1-h scan or >1.3 on a 3h scan [125]. Single-photon emission computed tomography enables a more accurate assessment of tracer uptake in the myocardium and blood pool, and it is recommended by all national and international societies [22,31,102,124,125]. Optimal methods to quantitate SPECT studies (with and without CT) are currently under investigation [131].

In a large multicentre study involving 1,217 patients, false positives almost exclusively resulted from uptake by AL amyloidosis [126]. Bone scintigraphy with a Perugini grade of two or three of myocardial uptake showed a high sensitivity of >99% for ATTR-CM but a lower specificity of 82–86%, given that a grade of 1 or 2 can be observed in patients with AL-CM [99]. However, if urine and serum tests are negative for AL amyloidosis, the specificity of the test increases to 100%. But the images obtained with the different agents are not identical, with HMDP seeming to have a very low false positive rate [126,132,133,134]. Uptake of amyloid can also be noted in patients with early ATTR-CM as well as with other subtypes of cardiac amyloidosis, such as serum amyloid A and apolipoprotein A1 [99]. In addition, other conditions may cause cardiac uptake on bone avid tracer cardiac scintigraphy or increased extra skeletal accumulation in the region of the heart and/or other soft tissues, causing potential diagnostic pitfalls in ATTR-related amyloidosis [135,136,137,138,139] (Supplementary Figure 1).

Currently, in the absence of histological data, ATTR-CM can be confidently diagnosed when a patient presents with a clinical phenotype that is associated with echo or CMR findings suggestive of amyloidosis, grade 2 or 3 tracer uptake in the heart on bone avid tracer scintigraphy, and the absence of detectable monoclonal immunoglobulin in the blood and urine using sensitive assays.

The comparative performance of the different tracers remains unclear as ^99 m^Tc-DPD and ^99 m^Tc-HMDP are currently most often used in Europe, and ^99 m^Tc-PYP is the predominant or exclusive tracer available in the United States, Canada, and Japan [140,141].

At present, there is no established value in repeating scans to evaluate disease burden in patients with ATTR-CM. However, the potential benefits of repeating scans have not been extensively studies in the context of current ATTR-CM treatment approaches [111].

###### 3.2.5.2. Positron emission tomography

Positron emission tomography (PET) scans with amyloid-specific tracers, such as ^11^C-Pittsburgh compound B (PiB) [142], ^18^F-florbetaben [143], and ^18^F-florbetapir [144], detect cardiac amyloidosis. Nevertheless the uptake occurs not only in ATTR amyloidosis tissues but also in AL amyloidosis [142,143,144]. PET imaging can simultaneously visualize the distribution of amyloid deposits in other organs [145,146]. The goal of PET is to have high fidelity to identify the type of amyloid fibril protein as well as to have a more quantitative method to assess the amyloid burden and response to treatment [111].

PET results have been shown to correlate to findings on echo, CMR, and cardiac scintigraphy. Myocardial tracer retention (MTR) had a positive correlation with apical sparing, E/e′, and wall thickness, along with a negative correlation with tricuspid annular plane systolic excursion and the left ventricular end-diastolic volume on echo [147]. PET showed a 94% concordance regarding the extent of affected myocardium on CMR. Cardiac scintigraphy (DPD) matched PET results in most patients [147]. Follow-up PET scans do not yet have a defined role in clinical practice.

#### 3.2.6. Cardiac biomarkers

N-terminal pro-brain natriuretic peptide (NT-proBNP) and high-sensitivity cardiac troponin T (cTnT-HS) are important diagnostic and prognostic biomarkers used to evaluate the severity of cardiac involvement in ATTR-CM patients and are included in staging systems [148,149,150]. Increased levels of these biomarkers indicate more severe cardiac involvement and poorer prognosis [151]. This topic will be described in further detail in the section ‘How to evaluate disease evolution and prognosis’.

Disproportionally increased NT-proBNP levels to the degree of HF are commonly observed in all forms of cardiac amyloidosis and are a ‘red flag’ for the condition, as well as increased levels of cTnT-HS.

### 3.3. ATTR and extracardiac manifestations

**Figure 7:**
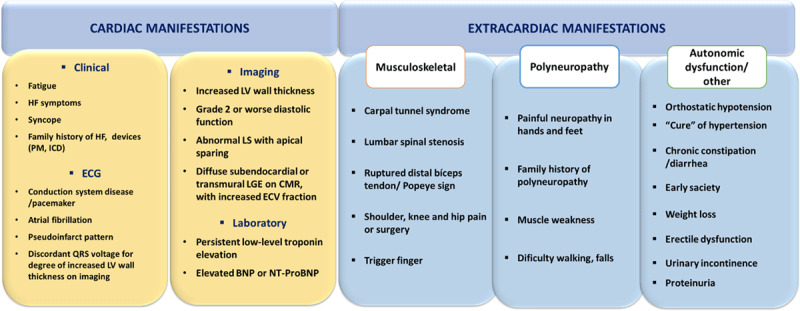
Clues (‘red flags’) to the presence of cardiac ATTR, Transthyretin amyloidosis; BNP or NT-proBNP, brain natriuretic peptides; CMR, cardiac magnetic resonance; ECG, electrocardiogram; ECV, extracellular volume; HF, heart failure; ICD, implanted cardioverter defibrillator; LGE, late gadolinium enhancement; LS, longitudinal strain; LV, left ventricle; PM, pacemaker.

In ATTR disease, besides cardiovascular involvement, other organs and systems are commonly affected (Figure 7). Neurologic manifestations like sensorimotor polyneuropathy, spinal stenosis, and carpal tunnel syndrome (CTS) as well as autonomic dysfunction with hypotension, urinary incontinence, sexual dysfunction, early satiety, and bowel alterations are frequent. Gastrointestinal involvement can lead to malabsorption; musculoskeletal issues may include weakness, fatigue, spontaneous biceps tendon rupture (Popeye’s sign), and degenerative joint disorders. Eye and auditory impairments also occur frequently [87]. These multisystem complications can cause functional decline, pain, fatigue, malnutrition, impaired fine motor skills, mobility problems, dependency and even social isolation and depression. Some of extracardiac features will be developed further in the section ‘Keys to an early diagnosis’.

### 3.4. How to suspect

#### 3.4.1. ‘Red flags’

There are several clinical contexts considered as high-risk scenarios for suspected ATTR-CM [80,152,153] and will be developed below. In the presence of any cardiac ‘red flag’ (Figure 7), clinical suspicion should arise with no specific age as a cutoff point. In addition, there are several extracardiac clues that may also point to the diagnosis [9] as previously mentioned.

#### 3.4.2 Keys to an early diagnosis

##### 3.4.2.1. Hig-Hrisk clinical scenarios

###### Heart Failure

Heart Failure with preserved ejection fraction (HFpEF) is a common condition affecting up to 50% of HF patients [4,5,6,154]. Among these, cardiac amyloidosis (mostly ATTR) may account for 6% to 30% of cases [8,68,69,71,155,156,157] with increased prevalence in those over 75 years of age.

While LVH is commonly observed in patients with ATTR-CM and HFpEF, in a study including HF patients with LV ejection fraction ≥50% and no LVH the prevalence of ATTR-CM was 5%, mainly affecting men over 80 years of age [158]. In patients with HF and reduced/mildly reduced ejection fraction, the prevalence of ATTR-CM was around 10% [159,160].

These findings highlight the importance of considering ATTR amyloidosis screening [31,140] in HF patients without an identified cause even in the absence of LVH, and in the presence of any value of LVEF.

###### Left ventricular hypertrophy

In patients diagnosed with LVH later in life, who are initially thought to have ‘sarcomeric’ HCM, it is crucial to search for other ‘red flags’ that may indicate cardiac amyloidosis. Cardiac amyloidosis is a condition that frequently mimics HCM, affecting 1% of HCM patients diagnosed between 40 and 48 years old, and 26% of those above 80 years of age [70]. It is important to note that sarcomeric HCM registries often include patients with a relatively high average age at diagnosis. Older patients with HCM more commonly have negative genetic studies in sarcomere genes [161,162]. In a series of 298 patients over 62 years of age with unexplained LVH and an initial diagnosis of HCM, testing for a TTR mutation revealed a 5% prevalence of ATTRv amyloidosis [163]. The study did not assess the presence of ATTRwt-CM [163].

###### Aortic stenosis

The coexistence of cardiac amyloidosis and AS is not uncommon in the elderly [82] and has therapeutic and prognostic implications [164]. Aortic valve infiltration by amyloid may contribute to the initiation and progression of AS [165], and in the last few years many studies investigated the association between these two diseases [23,67,72,166,167]. The prevalence of both, together, ranges from 6% and 16%, with higher prevalence in older male patients undergoing transcatheter aortic valve replacement (TAVR) for severe AS [23,67,72]. The prevalence is lower in surgical aortic valve replacement series involving patients over 65 years old [166,167].

Diagnosing associated cardiac amyloidosis, particularly ATTRwt-CM, may be challenging, as it shares common features with AS such LVH, diastolic dysfunction, and HF. However, the presence of low-flow low-gradient severe AS phenotype with mildly reduced ejection fraction is a ‘red flag’ for possible associated amyloidosis [23].

Due to the poorer prognosis with combined cardiac amyloidosis and AS, it is reasonable to screen elderly patients (>75 years) presenting with severe AS [67].

In a systematic review of prospective studies that performed bone avid tracer cardiac scintigraphy screening for TTR cardiac amyloidosis in different populations at risk, the authors identified 156 patients (11%) with ATTR-CM in the nine studies published between 2015 and 2020 and accounting for 1,375 screened patients. The prevalence varied in different settings and was particularly relevant in aortic stenosis (11%, mean age of 86 years), HFpEF (15%, mean age of 83 years), and LVH/HCM (13%, mean age of 75 years) [168]. The prevalence found in patients with CTS was 3% (mean age of 82 years). Considering the costs and efficacy of currently available targeted therapies for ATTR-CM, the authors question these screening settings for the early recognition of the disease [168].

###### Extra cardiac high-risk scenarios

Carpal tunnel syndrome, spinal stenosis, or spontaneous biceps tendon rupture are recognized ‘red flags’ for ATTR-CM (Figure 7) [75]. Carpal tunnel syndrome in particular, which primarily affects individuals over 50s has a prevalence of 15% to 60% in ATTR-CM [54,75,169] and can be a very early sign of the disease, manifesting several years before cardiac symptoms appear [170,171]. Notably, some patients may already show signs of cardiac involvement when undergoing CTS surgery [172]. In patients with ATTR-CM, the incidence rate of CTS is similar in genetic and sporadic forms [171].

When non-occupational CTS is bilateral, with symptoms recurrence after surgery and unexplained LVH is also present, the prevalence of ATTR-CM cardiac involvement is higher [173]. The concomitant presence of lumbar spine stenosis or spontaneous rupture of the biceps tendon are additional ‘red flags’ for ATTR-CM [174]. Thus, it is necessary to involve several surgical specialties (orthopaedics, neurosurgery, plastic surgery) in the early diagnosis of ATTR-CM.

##### 3.4.2.2. Incidental findings at the Echo laboratory

Echocardiographic ‘red flags’ for ATTR-CM can aid the early diagnosis of ATTR-CM. In a study with over 5,000 patients aged 55 or older undergoing echo examination, 7% exhibited at least one echo ‘red flag’ [174,175]. Among patients with the applied criteria, a multiparametric diagnostic algorithm revealed a 24% prevalence of ATTR-CM, increasing with age [174,176]. Patients with HF and unexplained LVH were commonly referred for echo examination, suggesting the echo laboratory may play a crucial role in identifying individuals suspected of having amyloid cardiomyopathy [175,176].

##### 3.4.2.3. Incidental detection of cardiac uptake in bone scintigraphy

The incidental finding of cardiac uptake of tracer on bone scintigraphy procedures performed for non-cardiac reasons may lead to an early diagnosis of previously unsuspected cardiac ATTR. However, it can also be a false positive or even correspond to a clinical situation other than ATTR [99,126,135,136,137,138,139,177,178,179] (Supplementary Figure 1). Thus, the incidental finding must always be interpreted in the appropriate context.

###### Is it common?

A review of 12,400 bone scans identified incidental myocardial uptake in 45 cases (0.36%) [180]. The prevalence increased with age and was more common in males [180]. In this and other recent studies [180,181,182], all patients with unexpected cardiac tracer uptake had evidence of LVH or increased indexed LV mass.

**Figure 8:**
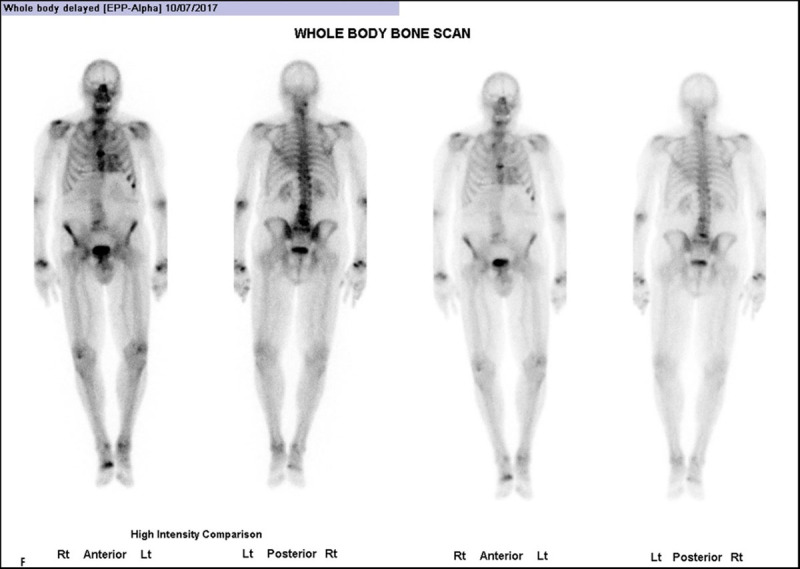
Whole body anterior and posterior planar views (shown at two intensities) performed 3 hours post-injection of Tc99 m HDP, investigating an 85-year-old man with back pain. He had no history of Heart Failure. (Images courtesy of Cabrini Health, Victoria, Australia).

**Figure 9:**
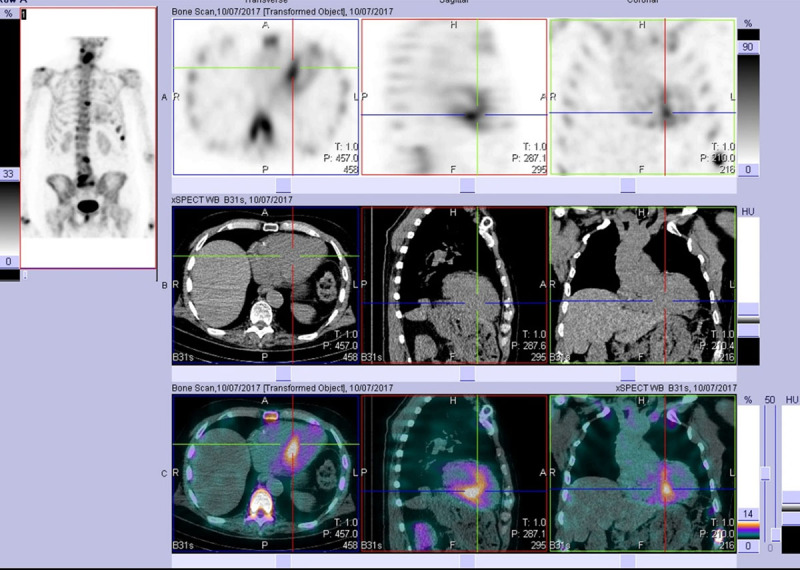
After viewing the planar study, a SPECT-CT was performed. This representative slice in axial, sagittal, and coronal plains (top row = SPECT, middle row = CT, bottom row = merged) confirms the uptake as cardiac and not blood pool. Echocardiogram at the time was normal. The patient developed echo and clinical features of cardiac ATTR amyloid approximately two years later. (Images courtesy of Cabrini Health, Victoria, Australia).

Similar findings were observed in a study of patients over 75 years old, with incidental myocardial uptake observed in 2.78% of cases [183]. The prevalence of cardiac uptake was higher (3.88%) in males compared to females (0.77%), especially in older age groups [183]. However, in some cohorts, the incidence of incidental cardiac uptake was lower. For example, a retrospective analysis in South Korea with ^99 m^Tc-DPD reported a prevalence of incidental cardiac uptake of 0.06%, but the study included only patients over 70 years of age [184]. Multiple case reports have highlighted the awareness of this condition among physicians [185,186]. Figures 8 and 9 show incidental cardiac uptake in a patient presenting for investigation of back pain. He was later confirmed to have cardiac ATTR amyloid.

A recent Australian study [182] of 6,918 patients found 15/3,472 ^99 m^Tc-HMDP positive scans, but only 1/3,446 ^99 m^Tc-MDP were positive. All HMDP-positive patients had increased septal wall thickness on echo. The heart/whole body ratio correlated positively with LV mass, and negatively with LV ejection fraction on echo. Not only did this group confirm the echocardiographic correlation, but they also suggested the well-appreciated poor sensitivity of ^99 m^Tc-MDP compared to other tracers.

These studies have highlighted that cardiac uptake on a bone scan occurs in certain subpopulations, particularly males and the elderly, with percentages ranging from 6% [182] to even 13% [183]. As progress in the diagnosis, it is important to acknowledge this group of asymptomatic patients who may present at amyloid clinics in the future.

To reduce false positives, performing SPECT or SPECT-CT is strongly recommended when incidental cardiac uptake is observed, as it provides better discrimination between myocardial retention from blood pooling compared to planar images [103].

###### What does it mean?

The incidental discovery of cardiac uptake in bone scintigraphy does not necessarily indicate a diagnosis of cardiomyopathy. However, as targeted therapies for ATTR-CM become available, early identification of cardiac amyloidosis becomes crucial in determining subsequent investigation strategies, such as thorough clinical assessment and potentially echo and/or CMR imaging. The specific steps to be taken should align with published recommendations and the diagnostic approach adopted by individual centres [187].

##### 3.4.2.4. Incidental detection of early ATTR cardiac disease with no evidence of cardiomyopathy

**Figure 10:**
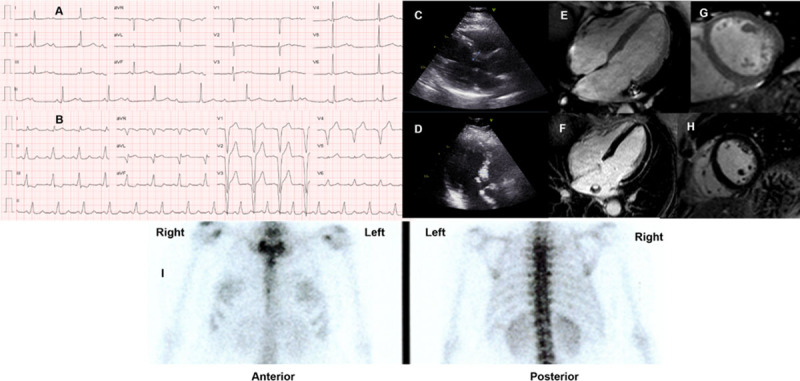
**(A)** ECG of a 48-year-old female (African ancestry), showing complete atrial-ventricular block on hospital admission after a syncope. She had mild hypertension, a history of paroxysmal atrial fibrillation, and right carpal tunnel syndrome. No known family history of amyloidosis. **(B)** ECG after pacemaker implantation. Echocardiographic images: **(C)** Parasternal longitudinal long-axis **(D)** Apical 3-chamber views, showing only mild and localized (basal septum) LVH (12 mm). Cardiac magnetic resonance **(E-H)** was also normal: no cardiac hypertrophy, and **(F, H)** no late gadolinium enhancement; **(I)** ^99 m^Tc-3,3-diphosphono-1,2 propanodicarboxylic acid (DPD) scintigraphy (chest images) showed no cardiac uptake three hours after radiotracer administration. Genetic testing identified the pathogenic mutation p.Val142Ile in the TTR gene (no other mutations in a large panel of genes studied by next-generation sequencing). Of her three children (adolescents), two have the mutation (no phenotype). (Images courtesy of Centro Hospitalar Universitário Lisboa Norte, Lisbon, Portugal).

Heart failure and LVH are late manifestations of ATTR-CM. In the absence of any of these conditions, an earlier diagnosis of cardiac ATTRv amyloidosis is clinically possible through the identification of unexpected isolated cardiac arrhythmias, particularly in younger patients (e.g., advanced AV blocks in patients under 50 years), even without clear findings on echo or CMR findings (Figure 10).

Documents issued by national or international societies regarding ATTR cardiac amyloidosis [22,31,102,124,125] showed a similar opinion regarding the indication for targeted treatment when there is clear evidence of cardiac disease on echo or CMR. However, gaps remain regarding the indication for treatment in specific scenarios, such as: 1) patients with positive bone scintigraphy in the absence of significant echo or CMR findings; 2) patients with significant cardiac arrhythmias and familial disease, but negative bone scintigraphy and no clear evidence of cardiomyopathy on echo or CMR exams (as shown in Figure 10); and 3) positive cardiac involvement in asymptomatic patients.

A study involving six international amyloid centres and including 118 patients with ATTR-CM (either genetic or wild type) [188] demonstrated that almost one-third of patients without HF symptoms at initial evaluation developed HF after a median follow-up period of 3.7 years. Treatment with TTR stabilizers was associated with less progression to clinical HF and improved survival [188].

### 3.5. How to diagnose

**Figure 11:**
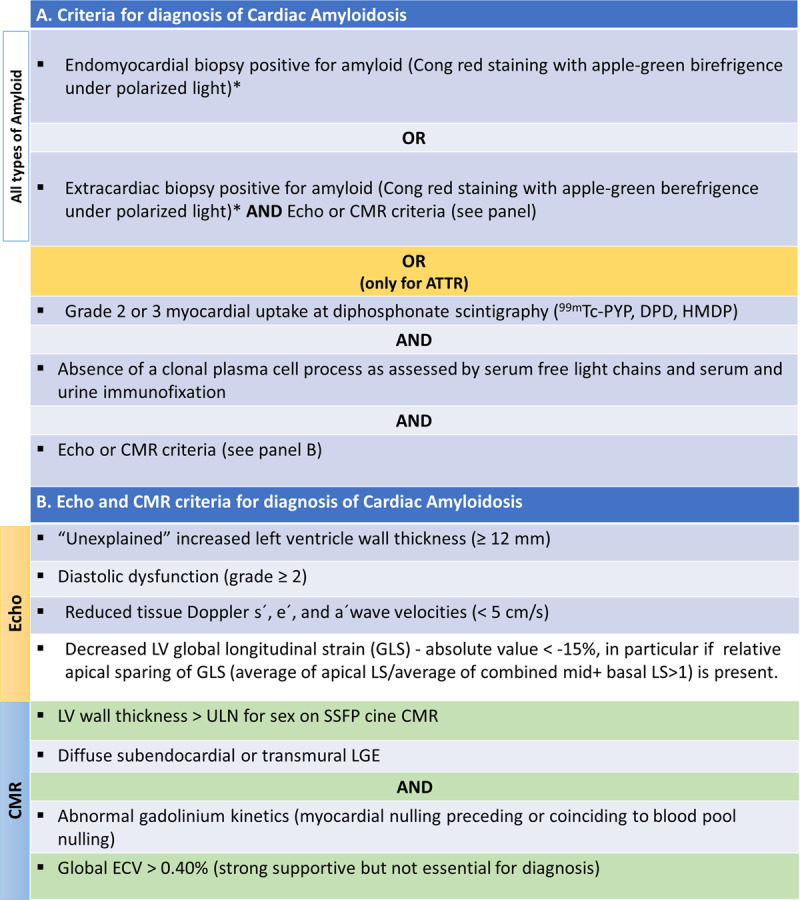
Diagnosis criteria of cardiac amyloidosis. **(A)** Criteria for diagnosis of cardiac amyloidosis. **(B)** Echocardiographic and cardiac magnetic resonance criteria for diagnosis of cardiac amyloidosis. Modified from [22,104]. *Classification of the amyloid fibril protein must follow (mass spectrometry or immunohistochemistry). ATTR, Transthyretin Amyloidosis; CMR, Cardiac magnetic resonance; Echo, echocardiogram; ECV, extracellular volume; LS, longitudinal strain; LGE, late gadolinium enhancement; LV, left ventricle; SSFP, steady-state free precession; ULN, upper limit of normal.

There are specific recommendations for the diagnosis of cardiac amyloidosis based on clinical and imaging findings and/or according to histological evidence [104]. Regarding ATTR-CM, the recommendations for diagnosis are shown in Figure 11.

#### 3.5.1. Pathways for the diagnosis

**Figure 12:**
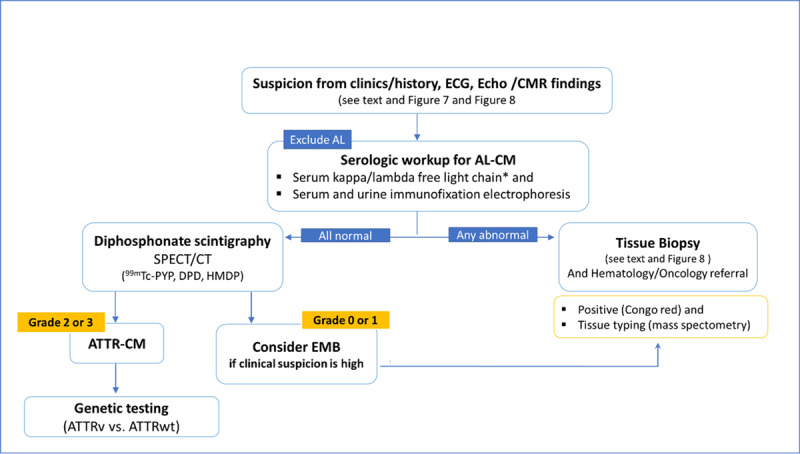
Algorithm for the diagnosis of Cardiac Amyloidosis. *Reference intervals for free light chain ratio according to renal function must be considered; AL-CM, Light chain cardiac amyloidosis; ATTR-CM, Transthyretin cardiac amyloidosis; ATTRv, genetic (hereditary) ATTR-CM; ATTRwt, wild type ATTR-CM; CMR, cardiac magnetic resonance; DPD, 3,3-diphosphono-1,2-propanodicarboxylic acid; Echo, echocardiography; EMB, endomyocardial biopsy; HMDP, hydroxymethylene-diphosphonate; K/L, kappa/lambda; PYP, pyrophosphate; ^99 m^Tc, technetium-99 m.

There are non-invasive and invasive diagnostic pathways for a diagnosis. The cardiac amyloidosis diagnostic algorithm (Figure 12) should begin with a monoclonal protein screen to assess the presence of a plasma cell disorder and, therefore, supportive evidence for AL-CM [189]. Although ‘bone’ scintigraphy has emerged as a cornerstone of non-invasive ATTR-CM diagnosis, cardiac uptake that is consistent with ATTR-CM (grade 2 or 3 uptake) may be present in over 10% to 30% of patients with AL-CM [126,190,191]. Thus, it is fundamental to choose the appropriate diagnostic pathway based on the presence or absence of a monoclonal protein [101,140,192]. A scintigraphy scan alone is neither appropriate nor valid to distinguish ATTR-CM from AL-CM [189].

##### 3.5.1.1. First step: exclude AL amyloidosis

AL amyloidosis can be essentially excluded by obtaining a monoclonal protein screen comprising 3 laboratory tests: serum free light chain (sFLC) assay, serum immunofixation electrophoresis (SIFE), and urine immunofixation electrophoresis (UIFE) [192,193]. If all tests are normal, AL amyloidosis has been excluded with a negative predictive value of >99% [102,194]. Serum/urine protein electrophoresis should not be used to exclude a monoclonal protein given its lower accuracy relative to immunofixation for AL amyloidosis. If any of the above three tests is abnormal, a non-invasive diagnostic pathway is no longer an option, and a biopsy of the involved organ is needed [9,31].

##### 3.5.1.2. Second step: if AL is excluded, proceed with the non-invasive pathway

If AL-CM is ruled out, bone avid tracer scintigraphy can be performed to assess for ATTR-CM. If this shows a grade 2/3, a diagnosis of ATTR-CM can be made, and genetic testing (TTR gene) must follow. If scintigraphy is negative or equivocal (grade 0/1), echo, CMR, and patient history need to be reviewed and referral for tissue biopsy should be considered for patients with ongoing clinical suspicion of cardiac amyloidosis.

##### 3.5.1.3. Genetic testing

Patients with a definitive diagnosis of ATTR-CM should undergo genetic testing (search for TTR gene mutations) to distinguish ATTRv amyloidosis from ATTRwt [22,31,102,124,125]. Genetic testing should be performed regardless of age [31,102], in concert with genetic counselling. It is worth mentioning that, in a recent study, 5% of individuals with ATTR-CM ≥70 years had ATTRv; among females, the prevalence was 10% [195]. Distinguishing genetic from wild-type disease assists not only with cascade testing of at-risk relatives but may also inform treatment strategy [189].

##### 3.5.1.4. Biopsy

Endomyocardial (EMB) biopsy should be performed (if another tissue biopsy does not confirm amyloid presence) in the following scenarios [31,189]: 1) high clinical suspicion of cardiac amyloidosis in a patient with a monoclonal protein by immunofixation electrophoresis and/or an abnormal sFLC K/L ratio; 2) high clinical suspicion for cardiac amyloidosis despite negative or equivocal scintigraphy; or 3) cardiac scintigraphy is unavailable [189].

While rare, EMB may also detect dual pathology of AL and ATTR-CM, further highlighting the importance of pursuing biopsy in the appropriate clinical context. Since cardiac amyloidosis is a diffuse process, the sensitivity and specificity of EMB approach 100% in both cases with adequate samples. However, in very early disease, amyloid deposition may be patchy, although this situation seems to be quite rare. If EMB is not possible, alternatives include fat pad biopsy (although, due to the low sensitivity, if it is negative it is not sufficient to exclude cardiac amyloidosis), renal biopsy (in patients with suspected renal amyloidosis), bone marrow biopsy, or, eventually, a biopsy of the lip, skin or digestive tract [125].

Once amyloidosis is identified on tissue biopsy, the tissue should then be typed with either mass spectrometry or immunohistochemistry to further determine the subtype [102].

### 3.6. Delayed diagnosis and misdiagnosis: facts and risks

Despite increased awareness and improved diagnostics, most patients with ATTR-CM experience a significant delay of about six years before receiving the diagnosis [35,36,101]. Factors contributing to this delay include symptom overlap with other conditions, low disease awareness, perception of it being rare, the historical need for invasive diagnosis (biopsy), and until recently, the lack of disease-modifying treatments [196].

A longer delay in diagnosis is observed when there is absence of a family history, a predominantly cardiomyopathy phenotype in ATTRv amyloidosis compared to patients with a mixed phenotype, who are diagnosed earlier [36,197], and when there is a history of CTS [196,198]. Misdiagnosis is common [13,36,199], particularly with hypertensive heart disease and HCM. Several consequences arise from a delayed diagnosis or misdiagnosis [36] (Table 1):

**Table 1:** Potential consequences arising from a delayed diagnosis or a misdiagnosis.


CONSEQUENCES OF DELAYED DIAGNOSIS OR MISDIAGNOSIS: SUMMARY KEY POINTS

Progression of cardiac involvement with more severe disease at diagnosis potentially reduces the efficacy of TTR stabilizing therapies [196,197].

Use of inappropriate treatment or diagnostic tests (invasive and/or non-invasive) for the misdiagnosed conditions [36,196]. – discomfort and unnecessary risks for patients – increased economic burden

Evaluation by multiple healthcare providers before ATTR-CM diagnosis – anxiety and distrust

Recurrent hospitalizations due to heart failure (common) – poor quality of life [26]

Delay in beginning a specific treatment for ATTR-CM, with the potential to change favorably the natural history and the prognosis.

- Progression of cardiac involvement with more advanced symptoms (higher NYHA class) and higher NT-proBNP values at diagnosis, as well as ECG markers of more advanced disease (prolonged PR interval, higher intraventricular conduction delays, and higher prevalence of AF) [197].
- Use of inappropriate treatment or diagnostic tests (invasive and/or noninvasive) for misdiagnosed conditions [36,196]. These may lead to discomfort and unnecessary risks for patients, and, in addition, represent an increased economic burden. In a series [24], as much as 43% of misdiagnosed patients were treated with beta-blockers and 26% with angiotensin-converting enzyme (ACE) inhibitors, treatments that may be poorly tolerated by ATTR-CM patients.
- Evaluation by multiple healthcare providers before an ATTR-CM diagnosis is established is common, and this uncertainty may cause patient anxiety and distrust. Also, recurrent hospitalizations due to HF are common, implying *per se* poor quality of life for ATTR-CM patients [26].
- Delay in offering patients a targeted treatment for ATTR-CM that can change favourably the natural history and the prognosis.

Advances in cardiac imaging and the availability of genetic testing have improved the diagnosis of ATTR-CM. Non-invasive diagnostic algorithms and consensus recommendations have the potential to increase disease awareness and lead to early diagnosis [11,140].

However, there are still gaps in awareness and diagnosis. Despite the growing utilization of bone scintigraphy as a diagnostic tool, the median time from symptom onset to ATTRwt amyloidosis diagnosis did not change over the past five years (>60 months from 2015–2019) [21].

Interestingly, the UK National Amyloidosis Centre reported a different scenario. A retrospective report of 1,967 with a confirmed diagnosis of cardiac ATTR over two decades showed improved survival due to increased diagnoses and referrals [200]. The use of bone scintigraphy and CMR imaging allowed for earlier diagnosis, resulting in decrease in the duration of associated symptoms before diagnosis. A higher proportion of patients were diagnosed at an early-stage disease with a less severe cardiac phenotype [200]. It is worth noting that the most diagnosed form of cardiac amyloidosis was ATTRwt. These data represent the natural history of cardiac ATTR, because nearly all patients with amyloidosis in the UK are evaluated at the National Amyloidosis Centre, and tafamidis, the only disease-modifying therapy approved for ATTR-CM, is widely unavailable in the UK due mainly to its high cost [201]. However, tafamidis is available in 64 countries all over the world and a delayed diagnosis represents a missed opportunity for timely treatment. In addition, an early diagnosis is crucial for effectively managing the serious cardiac condition that presents particularities in the treatment of HF, arrhythmias, and related comorbidities.

### 3.7. How to evaluate disease evolution and prognosis

It is necessary to evaluate the stage of ATTR-CM to predict prognosis and consider the indications for disease-modifying treatments. Biomarker-based prognostic staging systems have been developed for ATTR-CM. Two different staging systems have been proposed: one by Grogan et al., which utilizes NT-proBNP, and hsTnT plasma values in ATTRwt-CM [149] and other by Gillmore et al [150]. which combines NT-proBNP and the estimated glomerular filtration rate (eGFR) in both ATTRwt- and ATTRv-CM. The Gillmore staging system developed by UK National Amyloidosis Centre, bas been well validated [202]. It defines stage I as NT-proBNP ≤3000 ng/L and eGFR ≥45 ml/min, Stage III as NT-proBNP >3000 ng/L and eGFR <45 ml/min, and the remaining cases are considered Stage II. The median survival from diagnosis for patients with stages I, II, and III, was 69.2, 46.7, and 24.1 months, respectively [150]. Additionally, changes in NT-proBNP concentration during the first year of follow-up serve as a powerful independent predictor of mortality in ATTRwt-CM [203]. Imaging risk parameters such as ejection fraction, GLS, and CMR extracellular volume also may have prognostic value [98,204] but they have not been integrated into prognostic risk models. Staging systems primarily focus on prognosis rather than monitoring the disease course. To address this unmet need, a group of international experts in cardiac amyloidosis developed recommendations for monitoring the course of patients with ATTR-CM [205]. These recommendations include 11 measurable features across three separate domains: 1) clinical and functional endpoints, 2) biomarkers and laboratory markers, and 3) imaging and ECG parameters (Supplementary Table 2). One marker from each domain provides the minimum requirement for assessing ATTR-CM progression [205].

Monitoring for the development of ATTR-CM is recommended for individuals who are ATTRv mutation carriers with no phenotype yet [31,206] (Supplementary Table 2), as well as neurological, and ophthalmological evaluations every year when age approaches the predicted age-of-onset for the disease.

### 3.8. Treatment

To manage ATTR-CM, two main topics must be considered: 1) symptomatic therapy, including the treatment of cardiac symptoms, complications, and comorbidities; 2) disease-modifying therapy, acting on different phases of the amyloidogenic process with the aim of changing the natural history of the disease [31,207]. In addition, general supportive therapeutic measures can be very useful, helping the patient to achieve a better quality of life [208] (Table 2).

**Table 2:** Treatment of Transthyretin Amyloidosis Cardiomyopathy (ATTR-CM).


TREATMENT OF ATTR-CM: SUMMARY KEY POINTS

**Treatment of cardiovascular symptoms, complications and comorbidities**

Heart failure

Arrhythmias

Thromboembolism

Orthostatic hypotension

Correction of aortic stenosis and other valvular defects (if indicated)

Heart transplantation/other measures for advanced heart failure

**Specific treatments**

Transthyretin stabilizers

Transthyretin silencers

Other (possible non-genetic and genetic therapies)

**Supportive therapeutic measures**

Exercise & Nutrition

Vaccines

Individualized approach to physical and emotional patient’s needs


#### 3.8.1. Treatment of cardiac symptoms and complications

##### Heart failure

Managing HF in patients with cardiac amyloidosis is challenging because most drugs commonly used in patients with HF are not indicated or may have risks in patients with cardiac amyloidosis. Thus, effective treatment and management are more difficult, requiring unique considerations in treatment planning.

##### Diuretics

Congestion is a significant issue in patients with ATTR-CM and HF, and diuretics play a fundamental role in its control. High doses are often required, particularly in patients with severe diastolic or systolic dysfunction [209]. However, diuretic treatment should be administrated carefully to prevent excessive diuresis, which can lead to a significant decrease in preload with worsening renal function. Typically, a loop diuretic (e.g., furosemide or torsemide) is combined with a mineralocorticoid receptor antagonist (e.g., spironolactone, eplerenone). Caution must be taken when using diuretics in patients with autonomic dysfunction due to the risk of orthostatic hypotension.

##### Beta-blockers and calcium channel blockers

In general, restrictive cardiomyopathies, including amyloid cardiomyopathy, have limited tolerance to beta-blockers. This class of drugs can often cause low cardiac output, fatigue, conduction disturbances, hypotension, and even syncope in conditions with a restrictive physiology like ATTR-CM where heart rate significantly affects cardiac output [210]. Conversely, a notable clinical suspicion for possible cardiac amyloidosis is the development of profound hypotension and fatigue following the initiation of beta-blockers [211]. Similarly, non-dihydropyridine calcium channel blockers are poorly tolerated due to their negative inotropic, chronotropic, and dromotropic effects.

##### Angiotensin-converting enzyme inhibitors, angiotensin II receptor blocker and sacubitril/valsartan

There is currently no supporting evidence for the use of these drugs in patients with cardiac amyloidosis. Additionally, it should be noted that the tendency to hypotension can be exacerbated in the presence of autonomic dysfunction.

##### Digoxin

Traditionally, digoxin has been contraindicated in cardiac amyloidosis due to its potential to bind to amyloid fibrils, resulting in increased toxicity. However, a recent study concluded that if digoxin is considered necessary for rate control in patients with AF, it can be cautiously used in cardiac amyloidosis [212]. According to the study’s findings the authors suggested using low doses (0.125 mg/day or lower) while closely monitoring digoxin concentrations, renal function, and electrolytes levels.

##### Sodium-glucose cotransporter type 2 inhibitors (SGLT2is)

SGLT2is (also called ‘Gliflozins’) have a class I indication for patients with symptomatic HF [213]. In an observational study, the initiation of dapagliflozin was well tolerated in patients with ATTR-CM who were already receiving tafamidis treatment [214]. However, the efficacy of SGLT2is therapy in ATTR-CM patients requires further investigation through randomized controlled trials as the major trials involving SGLT2is excluded patients with known cardiac amyloid.

##### Antiarrhythmic treatment

Atrial arrhythmias, particularly AF, are commonly observed in cardiac amyloidosis, due to atrial dilatation and atrial wall infiltration with amyloid. Achieving rhythm and rate control can be challenging in these patients, as there are limited pharmacological options. Amiodarone is frequently prescribed and generally well tolerated [210]. Other drugs used in clinical practice include sotalol and dofetilide. For appropriately selected patients, catheter ablation for AF may be a viable strategy, yielding the best outcomes when performed earlier in the disease progression [215,216]. However, further data are needed to assess the efficacy of catheter ablation in ATTR-CM patients with AF. Ventricular arrhythmias are also common in the disease and amiodarone may be considered for pharmacological treatment [210]. Sustained ventricular tachycardias may pose a risk for sudden cardiac death [210].

##### Anticoagulant therapy

Intracardiac thrombosis is frequent in cardiac amyloidosis, even in the presence of sinus rhythm and preserved systolic function, likely due to poor atrial function [217]. This has a significant impact on mortality [217]. In the presence of atrial mechanical dysfunction, anticoagulation may be considered, even for patients with sinus rhythm [218,219]. The decision for anticoagulation should not be solely based on the CHA_2_DS_2_-VASc score, since no association was found between this score and the presence of left atrial appendage thrombus on transoesophageal echocardiogram (TEE) and embolic events [220,221,222]. Moreover, when cardioversion for atrial arrhythmias is planned, TEE should be performed to rule out left atrial thrombi even among patients who receive an adequate anticoagulation regime [219].

Anticoagulation with either warfarin or novel oral anticoagulants is generally safe for patients with cardiac amyloidosis [209,222]. However, in cases where there is a prohibitive bleeding risk, consideration may be given to left atrial appendage closure devices, as they may reduce bleeding complications and ischemic cerebrovascular events without increasing the rate of early or mid-term complications [223].

##### Pacemakers and cardiac defibrillators

Conduction disturbances and bradyarrhythmia are highly prevalent in cardiac amyloidosis and are associated with a high rate of pacemaker (PM) implantation during follow-up [224]. In patients with ATTRv amyloidosis and predominant neurologic involvement, cardiac involvement typically presents as arrhythmias and conduction disorders due to dysautonomia and amyloid deposition in the heart. Cardiomyopathy presenting as HF is less common in early forms of familial amyloid polyneuropathy. However, managing asymptomatic patients with evidence of conduction disorders remains challenging, and the follow-up should include serial ECG and Holter recordings [225,226,227]. In patients with ATTR-CM requiring pacing, biventricular pacing should be considered to minimize deleterious remodeling and progressive HF associated with higher burden of right ventricular pacing [228]. Implantable cardioverter defibrillators (ICDs) are recommended in cases of aborted sudden cardiac death (SCD) with an expected survival >1 year or significant ventricular arrhythmias [102]. However, the benefit of ICDs for primary prevention of SCD in patients with ATTR-CM is uncertain [229], as no clinical predictors of appropriate ICD therapy have been identified [230]. Guidelines differ in their recommendations for primary prevention of SCD: the 2023 American guidelines recommend an individual decision [189] and the European Society of Cardiology refers that it is usually not recommended [31].

##### Orthostatic hypotension

Autonomic dysfunction with orthostatic hypotension can be managed using medications that act as norepinephrine replacers such as midodrine (2.5 to 10 mg oral, t.i.d) or droxidopa (100 to 600 mg oral, t.i.d.). Non-specific treatments like fludrocortisone (0.1 to 0.2 mg once daily) or octreotide (12.5–25 mcg, up to 1 to 3 times/day, subcutaneous injection) can also be considered. However, these interventions may be poorly tolerated in patients with HF due to the potential for fluid retention [189]. An alternative option without the risk of fluid retention is pyridostigmine, and anticholinergic medication for orthostatic hypotension [231]. Nonpharmacological interventions such as wearing compression stockings, discontinuing hypotensive medications (i.e., tansulosin, carvedilol, clonidine, tizanidine, nitrates, sildenafil citrate and tricyclic antidepressants), and increasing the daily intake of water can also be beneficial.

##### Advanced HF and heart transplantation

The correction of valve defects (mitral/tricuspid regurgitation, aortic stenosis), when indicated, makes part of the supportive therapy that should be offered to ATTR-CM patients. The specific issue of aortic stenosis has been addressed previously.

In specific cases of ATTR-CM and refractory HF symptoms, advanced therapies and heart transplantation (HT), may be considered as an option [232]. The identification of suitable candidates for advanced HF therapies requires a comprehensive evaluation of various factors and include clinical assessment, laboratory testing, and imaging studies. This ensures that the patients who are most likely to benefit from these therapies are selected. Frailty is an important consideration that can impact outcomes and should be considered [233].

The use of durable LV assist devices as a bridge-to-transplant or as destination therapy [234,235] is a viable option for highly selected patients with cardiac amyloidosis, particularly in those without significant right ventricular dysfunction prior to implantation. Reasonable outcomes have been observed in such cases [235].

Certain contraindications exist for HT, including the extent of extracardiac involvement and its potential impact on post-transplant morbidity and mortality [189]. In the context of ATTR-CM, extracardiac involvement such as gastrointestinal (GI) involvement and autonomic neuropathy may be factors that contraindicate HT. Gastrointestinal involvement can lead to malnutrition, increasing the risk of infection and impairing wound healing. Additionally, pre-existing disabling neuropathy will not improve after HT and can significantly impair rehabilitation efforts and quality of life [189]. For ATTRwt-CM specifically, some centres may consider age of 70 or older as a potential barrier if significant extracardiac organ involvement is present. For ATTRv amyloidosis-CM, heart-liver transplantation has traditionally been considered in patients at risk of neuropathy since neuropathy can progress even after HT alone. However, currently, HT alone may be an option as TTR-specific therapy, such as tafamidis or a silencing agent (in patients with ATTRv-CM), can be prescribed post-transplant if coexisting amyloidosis-related polyneuropathy is present [236].

#### 3.8.2. Specific drug therapies in ATTR

##### 3.8.2.1. Transthyretin stabilizers

###### Tafamidis

Until a few years ago, ATTR-CM was considered a rare untreatable disease [22]. However, the approval of tafamidis, a TTR stabilizer, has changed the treatment landscape for this condition. Initially approved for early-stage ATTRv amyloidosis with polyneuropathy tafamidis demonstrated efficacy also for patients with ATTR-CM, both hereditary and wild type. In a landmark phase III trial called ATTR-ACT, tafamidis treatment [11], at oral doses of 80 mg or 20 mg once daily, showed reductions in all-cause mortality and cardiovascular-related-hospitalizations, and reduced the decline in functional capacity and quality of life compared with placebo [11]. The benefits of tafamidis were significant and consistently observed in functional capacity and health status, starting from six months and lasting up 30 months [11,237]. Another study indicated tafamidis treatment can improve exercise capacity, as defined by cardiopulmonary exercise testing, in approximately 50% of treated patients [238]. ATTR-ACT, and its open-label long-term extension trial demonstrated that tafamidis at the dosage of 80 mg provided greater survival benefits compared to 20 mg [239]. The survival benefit persisted in the long-term, with a median follow-up of 58 months [240].

Tafamidis slows the progression of the disease by stabilizing the native TTR tetrameric structure, thus preventing misfolding, fibril formation and cardiac deposition, but it does not necessarily result in the regression of cardiac deposits.

Currently, tafamidis, is the only approved medical therapy for ATTR-CM, including both ATTRv amyloidosis and ATTRwt at the dosages of 61 mg (free acid) or tafamidis meglumine 80 mg, once daily [11,22,31,102,124,240] Since its first approval in Japan in February 2019, tafamidis has been approved for this indication in 64 countries [22,31,102,124,125].

###### Considerations regarding treatment with tafamidis

The main factor for initiating treatment is whether the patient will experience significant benefits from it and if the medicine is accessible. Age is not a contraindication, but decisions for patients aged 90 years and older should involve individualized discussions [11,189]. Patients in NYHA functional class IV are not eligible for this therapy, while those in functional class I to III, preferably class I-II, are candidates as the expected benefit is greater in less advanced cardiac disease [22,31,102,124,125]. Other considerations for treatment eligibility include the degree of functional disability, measured using the 6-minute walking distance (<100 m considered a severe disability that may exclude indication for treatment) [130,241], expected survival (if less than two years, there is probably no indication for this therapy), and frailty. Associated severe uncorrected AS or significant kidney dysfunction (eGFR<25 ml/min/1.73 m^2^) may also be additional exclusion criteria for tafamidis treatment [130].

However, eligibility for treatment can vary between different countries and even between different institutions in the same country, leading to unfair access inequalities. The high price of tafamidis is another limiting factor [189,242], making it the most expensive cardiovascular medication listed [87,201,243].

In countries where tafamis is approved, barriers related to prescription and affordability exist [189,242]. Financial assistance (partial or total), whether from insurance companies foundations, or the manufacturer, is often required [189,242]. Some countries, such as Portugal and Spain, fully cover all costs related to tafamidis through their national healthcare system.

###### Other TTR stabilizers

- *Acoramidis* (AG10) is a selective oral drug that has been demonstrated to increase serum TTR levels and to be well tolerated in patients with ATTR-CM and symptomatic HF [244]. The drug is currently being tested in an ongoing phase 3 trial (ATTRibute-CM trial, NCT03860935).
- *Diflunisal* is a non-steroidal anti-inflammatory drug that binds to the thyroxine binding sites of TTR, preventing dissociation of the TTR tetramer and amyloid fibril formation [211]. Diflunisal is effective in ATTR polyneuropathy (not yet approved). Even though it has the potential to cause GI bleeding, renal dysfunction, fluid retention, and aggravation of HF, it can be well tolerated in selected patients with ATTR-CM [245], has shown favourable effects on cardiac structure and function [246], and may prolong survival [247].

##### 3.8.2.2. Transthyretin silencers

- *Patisiran* is an intravenously administered first-generation small-interfering RNA (siRNA) encapsulated in lipid nanoparticles. It inhibits the synthesis of both TTR wild-type and variant alleles by targeting TTR mRNA in the liver [248]. In the APOLLO trial [14], a phase 3 trial of patisiran in patients with ATTRv amyloidosis with neuropathy, patisiran improved multiple clinical manifestations leading to drug approval for the treatment of ATTRv amyloidosis with neuropathy by both the FDA (any stage of the disease) and EMA (Stages 1 and 2).

In the prespecified cardiac subpopulation comprising patients with a baseline left ventricular wall thickness ≥13 mm and no history of hypertension or aortic valve disease. Patisiran reduced mean LV wall thickness and LV mass, increased the LV end-diastolic volume, improved LV GLS, and showed lower values of NT-proBNP, compared with placebo. These findings suggested the drug might halt or even reverse the progression of ATTR-CM [249,250]. Additionally, a small study in patients with ATTRv amyloidosis, demonstrated that patisiran led to significant reduction in ECV as measured by CMR, along with clinical, functional, and scintigraphy benefits [251]. Recently, in the APOLLO-B trial, patisiran demonstrated a statistically significant benefit in functional capacity (measured by the six-minute walk test, as well as improvements in health status and quality of life, compared to placebo, in patients with ATTR-CM regardless of whether hereditary or wild type [252].

- *Vutrisiran* is a second-generation siRNA administered as a subcutaneous injection every three months. The drug showed promising results in an exploratory cardiac investigation (NT-proBNP, echocardiography, and scintigraphy) in the phase 3 HELIOS-A trial in patients with genetic ATTR with polyneuropathy [253,254]. The phase 3 HELIOS-B trial in patients with ATTRv amyloidosis or ATTRwt cardiomyopathy is ongoing (NCT04153149).
- *Inotersen* is an antisense oligonucleotide (ASO) for ATTRv amyloidosis that targets TTR mRNA and induces degradation, preventing RNA translation and expression of both wild-type and mutant TTR. The NEURO-TTR phase 3 trial established the efficacy of inotersen in patients with ATTRv amyloidosis-PN [255]. An open-label extension confirmed clinical stability and safety in the long term [256]. The drug is approved by FDA and EMA for the treatment of patients with ATTRv amyloidosis with mild to moderate (stages I and II) neuropathy. In patients with ATTR-CM, inotersen also showed efficacy and safety [257,258].
- *Eplontersen* is a second generation ASO designed to improve safety profile. The NEURO-TTRansform trial (NCT04136184), a phase III study, was designed to evaluate the efficacy and safety of the drug compared with an external placebo group in 168 patients with ATTRv-PN [259,260]. Patients enrolled had Coutinho stage 1 or 2 ATTRv-PN, a documented TTR sequence variant, and signs/symptoms of polyneuropathy. Overall, ~28% of patients enrolled in the trial also had ATTRv-CM. An interim efficacy analysis occurred at Week 35 and the final analysis occurred at Week 66 [260,261]. Results from the Week 66 final analysis showed that eplontersen halted progression of neuropathy impairment and significantly improved quality of life, compared with external placebo at Week 66 [261]. Eplontersen treatment resulted in significant and sustained reduction in serum TTR concentration from baseline compared with external placebo at Week 65 [261]. In addition, eplontersen was well tolerated and demonstrated an acceptable safety profile at Week 66 [261]. In patients with ATTR-CM, the CARDIO-TTRansform (NCT04136171), a phase III study, is assessing the efficacy and safety of eplontersen 45 mg compared with placebo in ∼1400 adult patients with genetic or ATTRwt-CM and a history of HF. The primary endpoint is a composite of cardiovascular (CV) mortality and recurrence of CV clinical events at study end. The study, which is estimated to be completed by 2025, also includes an imaging sub-study which will measure the amyloid burden over time in a subset of enrolled patients (NCT04136171).
- Another silencing approach for ATTR-CM uses CRISPR/Cas9 gene editing technology to reduce TTR levels [262,263] with promising results for managing ATTR-CM [264]. While reducing TTR levels, TTR silencers also remove the ability of TTR to act as a transport protein, and thus, vitamin A supplementation is required when using TTR silencers [265].

##### 3.8.2.3. Other specific therapies

Other therapies have been attempted in ATTR-CM, such as the use of TTR degraders with the combination of *doxycycline* and *tauroursodeoxycholic acid* (TUDCA) [266], or are being studied, including anti-monoclonal antibody targeting TTR deposits in patients with TTR cardiac amyloidosis (NCT04360434). In a phase I study, the safety and pharmacokinetics of ascending doses of the anti-monoclonal antibody NI006 (ALXN2220) for the depletion of TTR deposits (i.e., ATTR depleter) were assessed in patients with ATTRv-CM or ATTRwt-CM and chronic HF [267]. Cardiac imaging studies were also undertaken. The use of NI006 was not associated with drug-related serious adverse events. Cardiac tracer uptake on scintigraphy and ECV on CMR were reduced and median NT-proBNP and troponin T levels were also reduced [267].

#### 3.8.3. General Supportive Treatment

Supportive treatment is fundamental in managing this chronic disease, along with other appropriate forms to control the production and deposition of amyloid. Each patient requires a tailored treatment plan based on their specific needs, symptoms, limitations and disease stage. Following general guidelines can alleviate symptoms and enhance the patient’s well-being and quality of life [208].

##### Exercise & nutrition

Light or moderate-intensity exercises can help alleviate pain and fatigue in amyloidosis patients. Sleep therapy may be beneficial for those experiencing sleep-related fatigue.

Dietary guidelines include a reduced-salt diet in HF patients, small frequent meals, and refraining from coffee, alcohol, and spices, namely in the case of bowel symptoms. A balanced diet and adequate fluid intake are important.

The support of a physiotherapist and a nutritionist within a multidisciplinary team management is crucial in managing amyloidosis and is typically available at specialized amyloidosis centres.

##### Green tea

Patients often inquire about the potential benefit of ‘natural products’ such as green tea for amyloidosis. Some studies suggest that polyphenols in tea, consumed in high quantities, may help prevent amyloid protein aggregation and deposition, potentially impacting positively the disease [268,269,270]. In ATTR-CM patients the treatment with green tea extract for nine months reduced left ventricular wall mass and thickness in ATTR-CM patients [268]. However, a single-centre retrospective study showed no survival benefit in patients who received a 675 mg daily dose of EGCG (Epigallocatechin-3-gallate, a polyphenolic natural compound abundant in green tea) compared to those receiving symptomatic treatment alone [271].

##### COVID-19 Vaccine

Limited date is available on COVID-19 in patients with amyloidosis. Nonetheless, these patients are at higher risk for COVID-19 complications and mortality due to age, HF, other organ dysfunction, and comorbidities [272,273].

Although specific recommendations for amyloidosis patients are lacking, general indications form the World Health Organization’s should be applied, and vaccination is generally recommended unless contraindicated [274]. Support organizations like The UK National Amyloidosis Centre and the Australian Amyloidosis Network provide updated information and protective measures against COVID-19 for amyloidosis patients [275].

## 4. Palliative care

Palliative care focuses on improving the quality of life of individuals facing serious or life-limiting illnesses [276], including those with ATTR-CM. The literature including guidance to palliative care in amyloidosis is scarce. Nevertheless, as in other life-threatening disease, palliative care should be tailored to the preferences of the patient and caregivers, being initiated at any stage of the disease when any of the physical symptoms and/or emotional distress are interfering with the quality of life [189,276]. In addition, ATTR-CM patients are often older patients with other comorbidities, and polypharmacy is often an important issue to manage [189].

Here are some important aspects of palliative care that can be applied to patients with ATTR-CM:

- Symptom management [31,189,276,277]: ATTR-CM can cause a range of symptoms and signs due to HF; also, symptoms arising from other organ involvement may be troublesome. Palliative care can help manage these symptoms through medications, non-pharmacological interventions, and lifestyle changes.
- Advance care planning: palliative care can help patients with ATTR-CM plan for future medical care, including discussions about end-of-life care preferences and goals of care [278,279].
- Emotional and psychological support: palliative care can provide emotional and psychological support to patients with ATTR-CM and their families, helping them cope with the stress and uncertainty of living with a life-limiting illness.
- Spiritual care: palliative care can also address the spiritual needs of patients with ATTR-CM, including support for patients’ faith or belief systems.
- Care coordination: palliative care can help coordinate care among the patient’s healthcare team and other caregivers, ensuring that patients receive comprehensive and integrated care.
- Bereavement support: palliative care can provide bereavement support to patients’ families after their loved ones pass away, helping them cope with their grief and loss.

Palliative care specialists are often consulted only at the very end-of-life, but in fact, they shall make part of a multidisciplinary ATTR care team for frail and/or older patients, as palliative care can be provided at any stage of a life-limiting illness, including during active treatment [280,281].

## 5. The patient’s perspective

The hopes, fears and needs of those living with amyloidosis must be known and communicated to allow for effective interactions between patients, physicians and other healthcare professionals (Table 3). Amyloidosis Patients Associations and Amyloidosis Support Groups play a vital role in facilitating this interaction (Video 2).

**Table 3:** The patient’s perspective: what’s more important.


THE PATIENT’S PERSPECTIVE: SUMMARY KEY POINTS

**Pre-Diagnosis phase**

**To have an early and accurate diagnosis!**

**What’s necessary? To increase awareness of the disease**

Public campaigns (directed to the general population) Sensibilization of media Collaborative work: Amyloidosis support groups for patients, National Cardiology Societies, industry (sponsors)

Disseminate the diagnostic algorithms & Consensus documents and existing recommendations

**Diagnosis and post-diagnosis phases**

To have the facility of genetic testing

To get from his/her doctor full information about the disease (patients & family, and caregivers)

To be followed in centres with experience in managing amyloidosis/Excellence centres (Ecs)*

Straight relationship between Ecs and the physician’s doctor in the community

To get full information about treatment and prognosis, and discussion with the patient about its expectations


* Should include genetic counseling in case of hereditary transthyretin amyloidosis.

**Video 2:**
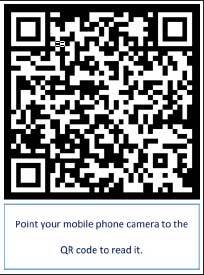
Testimonials of patients with amyloidosis. https://dulcebritocardiologista.com/wp-content/uploads/2023/01/Amyloidosis-The-Voice-of-the-Patient.mp4

Through online patient interviews (an initiative of national and international support groups for patients with amyloidosis, namely ATTR) [282], and published surveys results [283], the specific challenges faced by patients and doctors across different stages of the disease may be categorized as follows (based on authors’ opinion, experience, and clinical judgment):

### 5.1. Pre-diagnosis phase

One of the primary challenges is to achieve an early diagnosis of amyloidosis. Despite significant advancements in the understanding of ATTR-CM, including epidemiology, diagnosis, and treatment [21,26,199,283,284] there remains a substantial number of undiagnosed patients who have been observed by different specialists for several years before receiving the correct diagnosis [285,286,287]. This delay in diagnosis not only hampers timely treatment but also has profound emotional effects on patients [285,286,287].

Without targeted treatment, the survival rate after the development of HF is only three to five years [149,150]. However, specific therapy for both genetic and sporadic ATTR-CM has the potential to alter the course of the disease. Even in patients without initial HF symptoms, targeted treatment has shown to improve survival [188].

From the perspective of patients and quoting the President of the Austrian patient’s association ‘Living with Amyloidosis – Amyloidosis Austria’ (2023), personal communication, ‘[…] the delayed diagnosis and lack of appropriate treatment lead to insecurity, mental distress, and mistrust of different medical opinions. Time lost during this process is crucial and valuable in terms of patient’s lifetime’.

To address these issues and achieve earlier diagnosis, raising awareness about ATTR-CM is vital. This entails educating general practitioners, cardiologists, internal medicine specialists, neurologists, and other relevant specialists, as well as the general population, through the media and patient support associations. The World Amyloidosis Day, an awareness campaign initiated by the Amyloidosis Alliance [285], takes place on the 26th of October since 2021 and aims to reduce the time between symptoms onset and disease diagnosis [288].

Furthermore, disseminating established and published diagnostic algorithms particularly among the medical community, is important since many patients may initially seek care outside of specialized amyloidosis centres. Collaborative efforts between specialized centres, general practitioners, and patient associations are crucial throughout the entire process, involving educational activities and informational campaigns.

### 5.2. Diagnosis phase

Diagnosing amyloidosis and determining its specific type can be challenging.

Differences in diagnostic approaches and treatment options can vary across countries and even within the same country, according due to the variations in available tools, facilities, and the healthcare system organization. These discrepancies in diagnostic pathways and treatment choices may confuse patients if they lack sufficient information and guidance. Therefore, it is crucial for patients to be well informed about the disease’s characteristics, potential symptoms, available treatment options, disease progression, and prognosis. In the absence of adequate information from their physicians, patients may turn to the internet, which can potentially expose them to inaccurate or harmful information.

Following an amyloidosis diagnosis, testing the TTR gene is mandatory for the patients and their adult relatives, regardless of age or diphosphonate scintigraphy results. However, in many countries, genetic testing is not a routine practice outside of specialized centres [289].

Regardless of the clinical presentation, patients with ATTR disease face a chronic and life-threatening condition that significantly impacts their quality of life. However, there is a lack of published data on the patient’s journey and family experiences. The disease, whether primarily affecting the nerves or the heart, has been reported to be highly stressful for both patients and their families [290,291]. It leads to physical impairment, reduced quality of life, and decreased productivity, affecting patients and caregivers [283,290]. This highlights the importance of providing comprehensive information’s to patients and families, as understanding the disease can help optimize treatment opportunities.

A quality survey conducted at 15 selected amyloidosis centres in the US [283] involving cardiologists, nurses, patients, and patient advocates, revealed that the amount of time health professionals spent with patients was one of the most valued factors influencing their choice of amyloidosis centre for follow-up care.

### 5.3. Treatment phase

Patient’s access to specific treatment for ATTR-CM is a legitimate concern [283]. Currently, tafamidis is the only approved treatment and has broad access is most markets. However, there are differences in availability and reimbursement across countries. Some countries do not approve the treatment at all (e.g., UK), while others approve it but do not provide reimbursement (e.g., Brazil). In some cases, although the treatment is approved and reimbursed, the eligibility criteria for accessing medication may vary in different institutions even within the same country. Consequently, the number of patients worldwide who can access treatment may be more limited than desired. There is also a lack of consistent regulation among countries, including within the European Union.

Limitations on treatment access are primarily related to eligibility criteria and to the high cost of the drug. To note that not all patients with ATTR-CM are candidates for tafamidis. Patients in NYHA class IV, patients over 90 years old, and patients with multiple comorbidities limiting survival to less than two years, are not candidates. The reasons for these limitations need to be clearly explained to patients and their families. Disease staging systems for ATTR-CM [149,150] are not consistently used in clinical practice to determine eligibility for tafamidis treatment and may not considered factors like frailty, which can impact life expectancy [205,292]. Thus, a holistic approach is necessary, particularly in older patients [87].

Other patient concerns are related to the potential effectiveness of adjuvant therapies. Quoting again the president of the Austrian patients’ association ‘Living with Amyloidosis – Amyloidosis Austria’ (2023), personal communication: ‘[…] physicians are very worried about pharmacological treatment and do not value so much alternative or adjuvant therapies that might be particularly useful for patients and with impact on their quality of life, such as physical rehabilitation, dietary recommendations, mental/psychological treatment, and even meditation techniques’. Although there may not be large, randomized trials to test the efficacy of these activities, if patients experience added benefits, they should be given credibility’.

After diagnosis of genetic ATTR-CM, informing the family that they may have inherited a potentially fatal disease with significant life implications can cause significant distress. Genetic counselors should provide the necessary information and support, and a team effort involving psychological support for the patient and family may be required.

## 6. Patient advocacy and support groups

There are several websites and web channel initiatives of Amyloidosis support groups from many countries around the world, aiming at educating and empowering patients with amyloidosis [293,294]. The information and help provided by these patient support groups are invaluable and include recommendations on their websites regarding specialized amyloidosis centres, contacts, and information on the resources these centres offer. These include the possibility of participating in clinical trials, as well as dissemination of initiatives, meetings, educational programs, amyloidosis awareness booklets, animated videos with translation in several languages, interviews with scientific experts, and shared life-experiences with other patients with amyloidosis.

Closer cooperation between specialized centres and support groups for patients with amyloidosis should be increasingly nurtured, as it can be very advantageous for patients. It may even constitute a referral network for patients with HF to amyloidosis centres and thus contribute to an earlier diagnosis and care of ATTR-CM [283,285].

In addition, support groups may play a key role as a bridge between patients’ needs and healthcare policymakers.

## 7. Multidisciplinary approach

**Figure 13:**
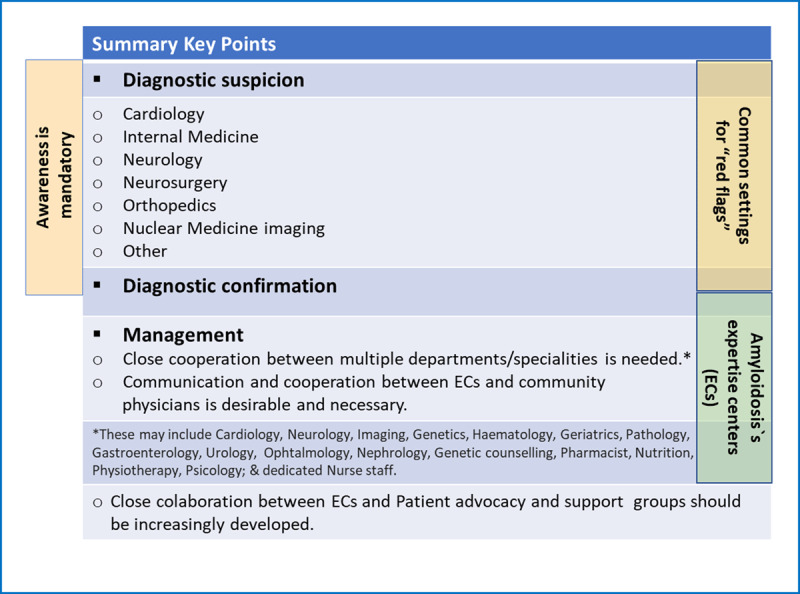
Transthyretin Amyloidosis: a multidisciplinary approach.

Amyloidosis requires a multidisciplinary approach for early diagnosis and effective management (Figure 13) [174]. While ATTRv amyloidosis was previously associated with neurological symptoms, it is now recognized that cardiomyopathy can be predominant [295]. Conversely, ATTRwt has traditionally been considered a cardiac disease [295] but tenosynovial and neurological problems are common [174,295]. In fact multiple organic systems may be affected and need supportive treatment.

Referral to specialized amyloidosis centres is recommended when suspicion of amyloidosis arises [283], although non-invasive diagnosis options facilitate earlier detection even outside of these centres [200].

### 7.1. The role of specialized amyloidosis centres

The multidisciplinary coordinated care that patients with ATTR amyloidosis need, with follow-up and support from different specialties, can be offered more easily in specialized centres. These centres exist in several countries, and play an integral role in assessing and treating amyloidosis, particularly ATTR-CM [296]. They offer comprehensive care, including expert staff, multidisciplinary approach, advanced diagnostics, patient education, and genetic counselling. They adhere to defined protocols and recommended diagnostic and therapeutic pathways [22,31,102,124,125,140]. In addition to regionality, international amyloidosis centres exist in diverse countries as Argentina, Australia, Brazil, Canada, France, Germany, Greece, Italy, Japan, the Netherlands, Spain, the United Kingdom (UK), and the United States [293,294].

### 7.2. Multidisciplinary coordinated care

Here is a summary of the contributions of the diverse specialties in providing coordinated care for patients with ATTR-amyloidosis:

1. **Cardiology:** Cardiologists are responsible for diagnosing, treating and monitoring cardiac abnormalities, such as cardiomyopathy and heart failure. They coordinate with other specialists (intervention cardiologists, electrophysiologists, cardiac surgeons), for advanced interventions like valvular repairs/replacements, implantation of cardiac devices or cardiac transplantation.
2. **Neurology:** Neurologists are involved in the care of patients with ATTR-amyloidosis who has neurological manifestations as peripheral neuropathy, autonomic dysfunction, and other neurological complications. All patients with ATTR-CM should be observed by a neurologist at the time of diagnosis and periodically thereafter.
3. **Ophthalmology:** An approach by ophthalmologists is important for ATTRv amyloidosis patients because severe ophthalmologic problems, such as vitreous opacity and glaucoma, are common [297], and almost all ophthalmologic disease manifestations are treatable either with surgery or with topical therapy [297].
4. **Nephrology:** Essential in the management of ATTR-amyloidosis patients who develop renal complications, such as proteinuria and renal dysfunction. Nephrologists monitor renal function, and may provide guidance on renal replacement therapies, including dialysis or kidney transplantation, if necessary.
5. **Gastroenterology:** Involved in the management of gastrointestinal manifestations of ATTR-amyloidosis which may include gastric problems, malabsorption and diarrhoea. Gastroenterologists provide nutritional support, and monitor liver function. In some cases, liver transplantation may be a therapeutic option for genetic ATTR.
6. **Haematology/Oncology:** Haematologists/oncologists are involved in the management of patients with AL-amyloidosis. In the diagnostic work up of ATTR-CM, haematologists may have a fundamental role in doubtful cases.
7. **Orthopaedics:** Orthopaedic surgeons or neurosurgeons may be involved in the management of patients with ATTR-amyloidosis who develop musculoskeletal complications, such as carpal tunnel syndrome or spinal stenosis. They assess and treat these conditions, which may include surgical interventions to relieve nerve compression or correct deformities.
8. **Urology:** Referral to urology may be necessary in case of urinary problems or incontinence, and geriatric and elder care medicine specialists may be of help particularly in case of older patients with multiple comorbidities, in whom frailty assessment may be decisive for planning therapeutic attitudes [87].
9. **Genetic Counselling:** Genetic counsellors play a crucial role in ATTR-amyloidosis, particularly for patients with hereditary forms of the disease. They provide genetic testing, evaluate the risk of disease transmission within families, and offer counselling regarding family planning options and the potential implications of the genetic diagnosis.

In addition to these medical and surgical specialties, a coordinated care team includes other healthcare professionals such as nurses, pharmacists, physiotherapists, nutritionists, psychologists, occupational therapists, and social workers.

A pharmacist is an important element of the multidisciplinary team, and several studies have demonstrated the effectiveness of such an approach to improve the overall quality of medication prescribing in older adults [298].

Dedicated nursing staff is of the utmost importance. Talking with the patient and informing the patient and family about the disease, what may be expected in terms of symptoms/signs, explaining treatment choices, and to discuss expectations, is a fundamental part in the teamwork.

Sharing information with patients regarding ongoing progress on amyloidosis research is important for many patients, and specialized centres often serve as a gateway for those interested in participating in clinical trials or seeking additional support through patient advocacy groups [283].

A multidisciplinary team contributes with their expertise to the comprehensive management of patients with ATTR-amyloidosis, aiming to improve outcomes and enhance the overall quality of life of these patients.

## 8 Challenges of ATTR-CM in low and middle-income countries

Transthyretin amyloid cardiomyopathy has been extensively studied in high-income countries, but the available research in low and middle-income countries (LMICs) may be more limited. There remains a significant gap in diagnosis in certain populations, and already published or ongoing randomized trials focused on patients with ATTR-CM, did not include African countries or low-income countries as recruitment centres [299]. However efforts are being made to improve awareness, diagnosis, and management of this condition globally [20,300,301,302,303]. ATTR-CM poses several challenges in LMICs:

- **Limited awareness and knowledge:** One of the primary challenges is the lack of awareness and knowledge about ATTR-CM among healthcare professionals in LMICs. This can result in underdiagnosis or misdiagnosis, leading to delayed or inadequate treatment.
- **Limited access to specialized diagnostic imaging:** Accurate diagnosis of ATTR-CM often requires specialized imaging techniques such as CMR imaging and nuclear scintigraphy. However, these imaging modalities may not be readily available or accessible in many healthcare settings in LMICs, making it challenging to confirm the diagnosis.
- **Cost and availability of genetic testing:** Genetic testing plays a crucial role in identifying specific mutations associated with ATTR-CM in the hereditary form of the disease. However, genetic testing can be expensive and may not be covered by healthcare systems in LMICs. Additionally, the availability of genetic testing facilities and expertise may be limited in certain regions.
- **Limited treatment options:** Treatment for ATTR-CM includes disease-modifying therapies, such as TTR stabilizers or, potentially, gene silencers, as well as symptomatic management of HF. However, access to these treatments may be limited or non-existent in LMICs due to factors such as high costs, lack of reimbursement, and regulatory challenges related to drug availability.
- **Healthcare infrastructure and resources:** LMICs often face resource constraints, including limited healthcare infrastructure, shortage of trained healthcare professionals, and inadequate healthcare funding. These challenges can hinder the timely diagnosis, management, and follow-up of patients with ATTR-CM.
- **Socioeconomic factors:** The socioeconomic factors prevalent in LMICs, such as poverty, limited access to healthcare services, and competing healthcare priorities, can further impact the diagnosis and treatment of ATTR-CM. Affordability of medications, transportation to specialized centres, and patient adherence to treatment plans can be significant challenges.

Addressing these challenges requires collaborative efforts between healthcare professionals, policymakers, and researchers to improve awareness, diagnostic capabilities, and treatment access for ATTR-CM in LMICs. Increased investment in healthcare infrastructure, training programs, and research initiatives can help mitigate these challenges and improve outcomes for patients with ATTR-CM in low and middle-income countries.

## 9. Conclusions

Transthyretin amyloid cardiomyopathy (ATTR-CM) has been increasingly recognized in various clinical settings. Its apparent higher prevalence is attributed to a growing interest, as it can be diagnosed non-invasively in most cases. In addition, effective therapy is already available and continues to evolve, improving patients’ quality of life and survival. Early diagnosis is crucial for better treatment outcomes. Clinicians should consider ATTR-CM in high-risk clinical settings for early diagnosis and intervention. However, access to treatment varies among countries, presenting barriers that must be addressed and overcome. Standardized treatment eligibility based on scientific evidence and on a holistic approach is needed, as not all patients are candidates for the same targeted therapies. Affordability represents a major challenge and globally may limit access to treatment for many patients. Negotiations involving insurers, foundations, manufacturers, and health regulatory bodies are needed to ensure fair financial assistance for these drugs. Investments in scientific and technological development in this area are particularly justified if they are used to improve the treatment of patients with ATTR-CM because heart involvement is the main determinant of prognosis.

## Additional File

The additional file for this article can be found as follows:

10.5334/gh.1262.s1 Supplementary Files. Figure s1 and Tables s1 to s2.
