# Supplementary material for: World Heart Federation Consensus on Transthyretin Amyloidosis Cardiomyopathy (ATTR-CM)
Source: Glob Heart. 2023 Oct 26;18(1):59. doi: 10.5334/gh.1262 (PMC10607607; doi:10.5334/gh.1262)
Supplement: Supplementary Files. — Figure s1 and Tables s1 to s2. [file gh-18-1-1262-s1.zip › s1-gh-1262_brito/WHF_ATTR_CM Supplementary Table 1.docx]

***Table S1*** *–* Survey on health services´ availability and accessibility related to cardiac amyloidosis.

| **Question** | | **%** |
| --- | --- | --- |
| **What is your role?** | |  |
| Clinician in Practice | | 63.4 |
| Clinical Scientist | | 2.4 |
| Head of Medical Department | | 19.5 |
| Clinical Researcher | | 4.9 |
| NGO representative | | 2.4 |
| Other | | 7.3 |
| **Are you completing this survey as a …** | |  |
| Cardiologist | | 70.7 |
| Internal Medicine Specialist | | 7.3 |
| Allied Health Professional | | 2.4 |
| Other | | 19.5 |
| **Do you think physicians in general are fully aware of what cardiac amyloidosis is?** | |  |
| Yes | | 29.3 |
| No | | 70.7 |
| **Do you know how to suspect cardiac amyloidosis?** | |  |
| Yes | | 90.2 |
| No | | 9.8 |
| **Have you seen and treated patients with cardiac amyloidosis? (Please indicate how often)** | |  |
| None | | 17.1 |
| 1 to 9 per year | | 63.4 |
| 10 to-19 per year | | 12.2 |
| More than 20 per year | | 7.3 |
| **In your country, do you have local or regional centers for the detection, diagnosis, treatment, and monitoring of patients with cardiac amyloidosis (eg cardiomyopathy consultation, amyloidosis referral centers)?** | |  |
| Yes | | 48.8 |
| No | | 51.2 |
| **Do you know what transthyretin cardiac amyloidosis (ATTR-CM) is?** | |  |
| Yes | | 90.2 |
| No | | 7.3 |
| **According to published literature, what proportion of patients with Heart Failure and a preserved ejection fraction do you think may have ATTR-CM as an underlying cause?** | |  |
| <10% | | 31.7 |
| 10% to 20% | | 61.0 |
| 20% to 40% | | 4.9 |
| >40% | | 0.0 |
| **Do you consider age as a potential red-flag for ATTR-CM?** | |  |
| Yes | | 85.4 |
| No | | 14.6 |
| **According to published literature, what proportion of elderly patients with calcified aortic stenosis do you think may have associated ATTR-CM?** | |  |
| <10% | | 19.5 |
| 10% to 20% | | 65.9 |
| 20% to 40% | | 12.2 |
| >40% | | 0.0 |
| **Do you know the importance of an early diagnosis of ATTR-CM?** | |  |
| Yes | | 95.1 |
| No | | 4.9 |
| **Are you aware that ATTR-CM is now potentially treatable?** | |  |
| Yes | | 82.9 |
| No | | 17.1 |
| **In your country what is the waiting time to see a general practitioner in the public health system as an outpatient?** | |  |
| Equal to or less than 3 months | | 92.7 |
| Equal to or less than 6 months | | 7.3 |
| **In your country what is the waiting time to see a cardiologist in the public health system as an outpatient?** | |  |
| Equal to or less than 3 months | | 80.5 |
| 4 to 6 months | | 17 |
| Other | | 2.4 |
| **Do you think an ECG may alert to a diagnosis of cardiac amyloidosis?** | |  |
| Yes | | 92.7 |
| No | | 7.3 |
| **What is the general waiting time for an echocardiograph (Echo) in the public health system?** | |  |
| Equal to or less than 3 months | | 80.5 |
| 4 to 6 months | | 14.6 |
| Other | | 4.9 |
| **If you consider an echocardiogram is suspicious for cardiac amyloidosis, which test would you order next (you can choose more than one option)** | |  |
| Cardiac magnetic resonance | | 63.4 |
| Nuclear medicine | | 68.3 |
| Cardiac biopsy | | 26.8 |
| Light-chains quantification | | 61.0 |
| Other | | 14.6 |
| **Typically, at what point in the care process is the presence of cardiac amyloidosis suspected?** | |  |
| Primary care - community health care, general practice | 12.2 | |
| Secondary care- medical care that is provided by a specialist  or facility upon referral | | 56.1 |
| Tertiary care– medical care by specialists working in a center that has personnel and facilities for special investigation and treatment | | 31.7 |
| **Typically, at what point in the care process is the presence of cardiac amyloidosis diagnosed?** | |  |
| Primary care - community health care, general practice | | 2.4 |
| Secondary care - medical care that is provided by a specialist  or facility upon referral | | 34.1 |
| Tertiary care – medical care by specialists working in a center that has personnel and facilities for special investigation and treatment | | 63.4 |
| **Do health professionals have appropriate and timely access in the cardiology department of a public hospital to tests and equipment for the diagnose of cardiac amyloidosis?** | |  |
| Yes | | 65.9 |
| No | | 34.1 |
