# Supplementary material for: World Heart Federation Consensus on Transthyretin Amyloidosis Cardiomyopathy (ATTR-CM)
Source: Glob Heart. 2023 Oct 26;18(1):59. doi: 10.5334/gh.1262 (PMC10607607; doi:10.5334/gh.1262)
Supplement: Supplementary Files. — Figure s1 and Tables s1 to s2. [file gh-18-1-1262-s1.zip › s1-gh-1262_brito/WHF_ATTR-CM Supplementary Figure 1.docx]

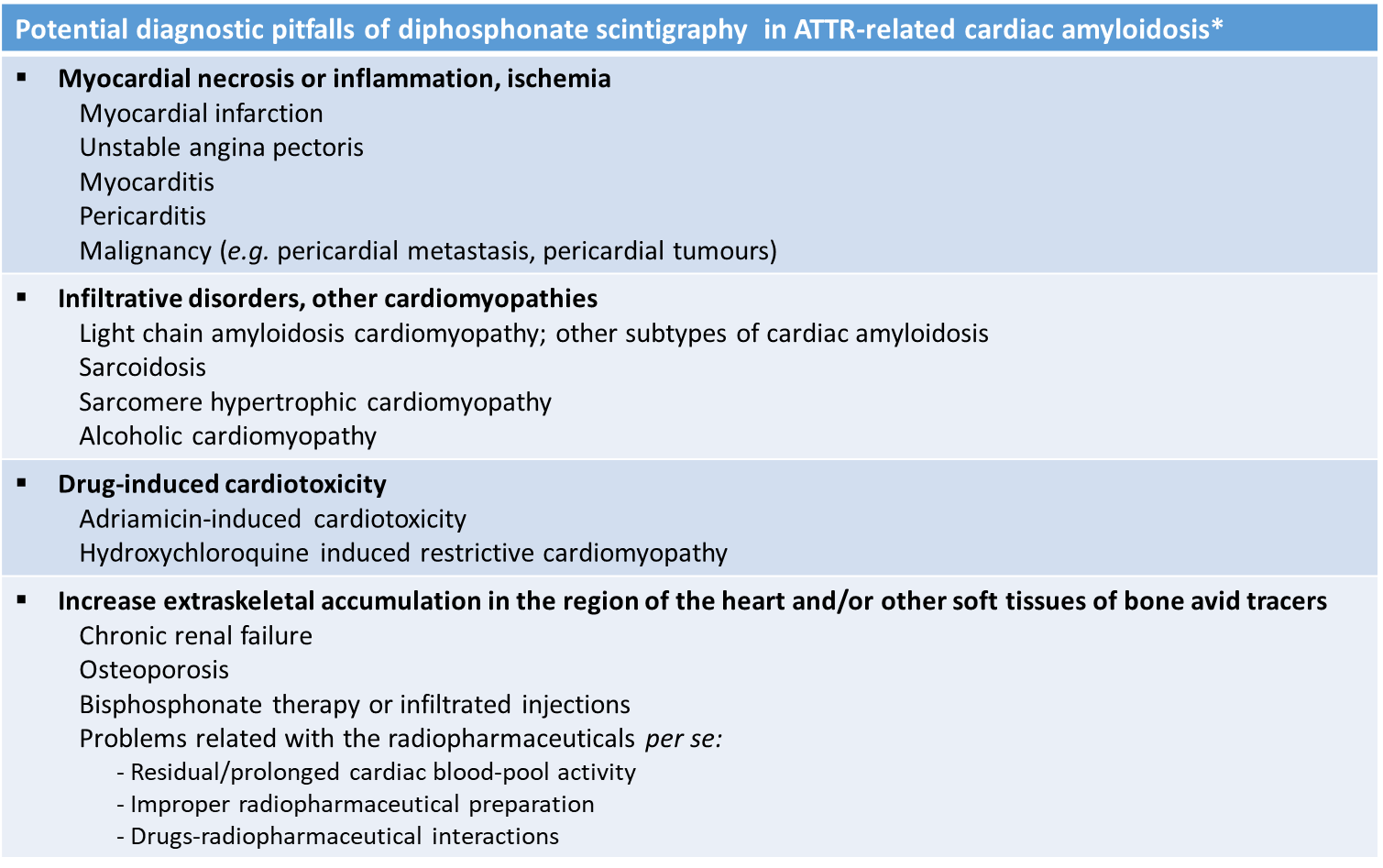
**Figure S1** – Potential diagnostic pitfalls of ^99m^Tc-labeled phosphate-based bone avid radiotracers (“bone” scintigraphy) in transthyretin-related amyloidosis. ATTR, Transthyretin amyloidosis. *The figure refers to false positive results. However false negative scans can also occur for certain ATTR genetic subtypes, and in patients with early transthyretin ATTR-cardiac amyloidosis ^135-139^
