# Supplementary material for: World Heart Federation Consensus on Transthyretin Amyloidosis Cardiomyopathy (ATTR-CM)
Source: Glob Heart. 2023 Oct 26;18(1):59. doi: 10.5334/gh.1262 (PMC10607607; doi:10.5334/gh.1262)
Supplement: Supplementary Files. — Figure s1 and Tables s1 to s2. [file gh-18-1-1262-s1.zip › s1-gh-1262_brito/WHF_ATTR-CM Supplementary Table 2.docx]

**Table S2:** Recommended measurement tools for evaluation of transthyretin amyloid cardiomyopathy course.

| **Tool and domain** | **Clinical feature** | **Threshold indicating disease progression** | **ATTR-CM Patients Recommended frequency of measurement** | **ATTRv Carri~~ers~~***  **Recommended frequency of measurement** |
| --- | --- | --- | --- | --- |
| **Clinical and functional** | | | | |
| **Clinical & medical history** | CV hospitalization | Any hospitalization by decompensated HF in a 6-month period | 6 months | 12 months |
| **NYHA class** | Change in class status (plus or minus indicate progression or improvement) | One class increase (note: must be measured during a 30-day period of stability) | 6 months | 12 months |
| **QoL: EQ-5D tool and KCCQ** | Description of measurements | Five-point decrease in KCCQ represents deterioration; 10% decline in EQ-5D score represents deterioration | 6-12 months | Not applicable |
| **Functional capacity** | 6MWT | Decrease of 30 – 40 m every 6 months | 6 months | Not applicable |
| **Laboratory biomarkers** | | | | |
| **Biomarker** | NT-proBNP | 30% increase with 300 pg/mL cut-off To be measured during a 30-day period of clinical stability and under same atrial rhythm | 6 months | 12 months |
| **Biomarker** | Troponin (high-sensitivity) assay | 30% increase | 6 months | 12 months |
| **Biomarker risk models** | Clinical staging system | Advance in NAC staging score | 6 months | 12 months |
| **Imaging parameters and ECG** | | | | |
| **Echocardiography or CMR** | LV measures wall thickness/mass | ≥2-mm increase in LV wall thickness | 6-12 months | 12 months (echo)  Every 3 years (CMR) |
|  | Systolic function measurements | ≥5% decrease in LV ejection fraction; ≥5 mL decrease in stroke volume; or ≥1% increase in LV global longitudinal strain by echo | 12 months |  |
|  | Diastolic dysfunction worsening | Stepwise increase in diastolic functioning grade; consistent deterioration in diastolic function by echo | 12 months |  |
| **ECG/Holter ECG** | New-onset of arrhythmic/conduction disturbances | New-onset bundle branch block New-onset AV block (of any degree) Sinus pauses, sinus node dysfunction, AF with a very slow ventricular response without pharmacologic treatment (<50 bpm) | 6 months | ECG every 12 months  Holter every 2 years |
| **Scintigraphy**  **(PYP, DPD or HMDP)** | Not applicable for FU | Not applicable for FU | Not applicable for FU | Every 3 years |

*Genetic council is offered to all vATTR subjects. AF= atrial fibrillation; ATTR-CM= transthyretin amyloidosis with cardiomyopathy; ATTRv= hereditary transthyretin amyloidosis; AV= atrioventricular; bpm= beats per minute; CM= cardiovascular magnetic resonance, CV= cardiovascular, DPD= ^99m^ Tc-3,3-diphosphono- 1,2-propanodicarboxylic acid) scintigraphy, ECG= electrocardiogram; echo= echocardiogram; EQ-5D= Health Questionnaire; FU= follow up; HF= heart failure; KCCQ, Kansas City Cardiomyopathy Questionnaire; HMDP = ^99 m^ Tc-hydroxymethylene diphosphonate scintigraphy, LV= left ventricle, NAC= UK National Amyloidosis Centre; NT-proBNP= N-terminal pro-B-type natriuretic peptide; PYP= ^99m^ technetium pyrophosphate (99 mTc-PYP) scintigraphy, Q-5D = EuroQol five dimensions; 6MWT= Six Minute Walk test. Adapted from ^36,98,204,205^
